# Methodological guidelines for P2X receptor assays and data interpretation

**DOI:** 10.1038/s41419-026-08730-0

**Published:** 2026-04-20

**Authors:** Dariusz C. Gorecki, Elena Adinolfi, Sahil Adriouch, Robson Coutinho-Silva, Tobias Engel, Flóra Gölöncsér, Friedrich Haag, Peter Illes, Kenneth A. Jacobson, Friedrich Koch-Nolte, Steven E. Mansoor, Carlos Matute, Gerry Melino, Ivana Novak, Anna Pegoraro, Pablo Pelegrin, Mauro Piacentini, Simon C. Robson, Robin M. H. Rumney, Michel Seman, Ronald Sluyter, Beáta Sperlagh, Yong Tang, Mario Tarantini, Henning Ulrich, Valerie Vouret-Craviari, Qing Ye, Gennady G. Yegutkin, Alexei Verkhratsky

**Affiliations:** 1 School of Medicine, Pharmacy and Biomedical Sciences, University of Portsmouth, Portsmouth, UK; 2 Department of Medical Sciences, University of Ferrara, Ferrara, Italy; 3 Univ Rouen Normandie, INSERM, U1234, Pathophysiology Autoimmunity and Immunotherapy (PANTHER), Normandie Univ, Rouen, France; 4 Laboratório de Imunofisiologia, Instituto de Biofísica Carlos Chagas Filho, Universidade Federal do Rio de Janeiro, Rio de Janeiro, Brazil; 5 Department of Physiology & Medical Physics, RCSI University of Medicine and Health Sciences, Dublin, Ireland; 6 FutureNeuro Research Ireland Centre, RCSI University of Medicine and Health Sciences, Dublin, Ireland; 7 Laboratory of Molecular Pharmacology, HUN-REN Institute of Experimental Medicine, Budapest, Hungary; 8 Institute of Immunology, University Medical Center Hamburg-Eppendorf, Hamburg, Germany; 9 Rudolf Boehm Institute for Pharmacology and Toxicology, University of Leipzig, Leipzig, Germany; 10 Molecular Horizons and School of Science, University of Wollongong, Wollongong, NSW Australia; 11 Molecular Recognition Section, Laboratory of Bioorganic Chemistry, NIDDK, National Institutes of Health, Bethesda, MD USA; 12 Department of Chemical Physiology & Biochemistry; Division of Cardiovascular Medicine, Knight Cardiovascular Institute, Oregon Health & Science University, Portland, OR USA; 13 Laboratory of Neurobiology, Universidad del País Vasco (UPV/EHU), CIBERNED-Instituto Carlos III, IIS-Biobizkaia, Department of Neurosciences, School of Medicine and Nursing, Leioa, Spain; 14 University of Rome “Tor Vergata”, Rome, Italy; 15 Department of Biology, Universitetsparken 13, University of Copenhagen, Copenhagen Ø, Denmark; 16 Department of Biochemistry and Molecular Biology and Immunology, Faculty of Medicine, University of Murcia, BioMedical Research Institute of Murcia, Murcia, Spain; 17 Departments of Anesthesia and Medicine, CLS 612, Beth Israel Deaconess Medical Center, Harvard Medical School, Boston, MA USA; 18 Bone and Joint Group, School of Human Development and Health, Faculty of Medicine, University Hospital Southampton, University of Southampton, Southampton, England; 19 International Joint Research Centre on Purinergic Signalling, School of Health and Rehabilitation, Chengdu University of Traditional Chinese Medicine, Chengdu, China; 20 Departamento de Bioquimica, Instituto de Quimica Universidade de Sao Paulo, Sao Paulo, Brazil; 21 Université Côte d’Azur, CNRS, UMR7284, INSERM, U1081, IRCAN, Nice, France & IHU RespirERA, Nice, France; 22 MediCity Research Laboratory and InFLAMES Flagship, University of Turku, Turku, Finland; 23 Faculty of Biology, Medicine, and Health, The University of Manchester, Manchester, UK

**Keywords:** Neuroscience, Ion channels in the nervous system

## Abstract

P2X receptors (P2XR) are a family of seven cation channels gated by extracellular ATP (eATP). Activation of P2XRs results in diverse cellular responses, including cell signalling, proliferation, differentiation, and death—all critically important in multiple physiological and pathophysiological states. These receptors, therefore, represent therapeutic targets of considerable interest. However, P2XRs, while structurally related, exhibit highly divergent and context-dependent functions. Their spatiotemporal and functional complexity is evident by overlapping expression across multiple cell types that can shift dynamically during physiological processes or disease progression. Furthermore, P2XRs can assemble as homo- or hetero-trimers, with distinct functional properties. These factors complicate definitive identification of a given P2XR responsible for a specific pathophysiological effect. Receptor activity in vivo is transient because of receptor-specific mechanisms and follows eATP breakdown by ectonucleotidases. Any correlation of ATP release with receptor engagement, as assessed in vitro, often does not correspond with the in vivo dynamics. Translation from animal models to humans is complicated by the species-specific pharmacology of some P2XRs, confounded by many animal models in use not fully replicating human P2XR function and regulation in pathology. Furthermore, there are no clinical biomarkers to distinguish incomplete receptor blockade from lack of therapeutic effect. Thus, translation has been very limited. To identify and validate specific P2XR functionalities, future experimental designs should use approaches and assays that can reliably assess receptor involvement, while reducing methodologically flawed findings. We propose guidelines developed in consultation with the purinergic community for consistent and reliable research practices in P2XR studies.

## FACTS

- Definitively determining which P2XR subtype mediates a given physiological or pathological effect remains challenging.
- Although heteromeric P2XR differ functionally from homotrimers, their physiological and pathological relevance is still poorly understood.
- Rapid eATP degradation and receptor-specific activation kinetics make it difficult to link ATP release to actual P2XR engagement in vivo.
- Differences between human and rodent P2XR pharmacology, coupled with the scarcity of human-relevant disease models, significantly complicate translational efforts.
- The absence of reliable biomarkers to distinguish insufficient P2XR blockade from genuine therapeutic ineffectiveness hinders clinical progress.

## Foreword

**Fig. 1:**
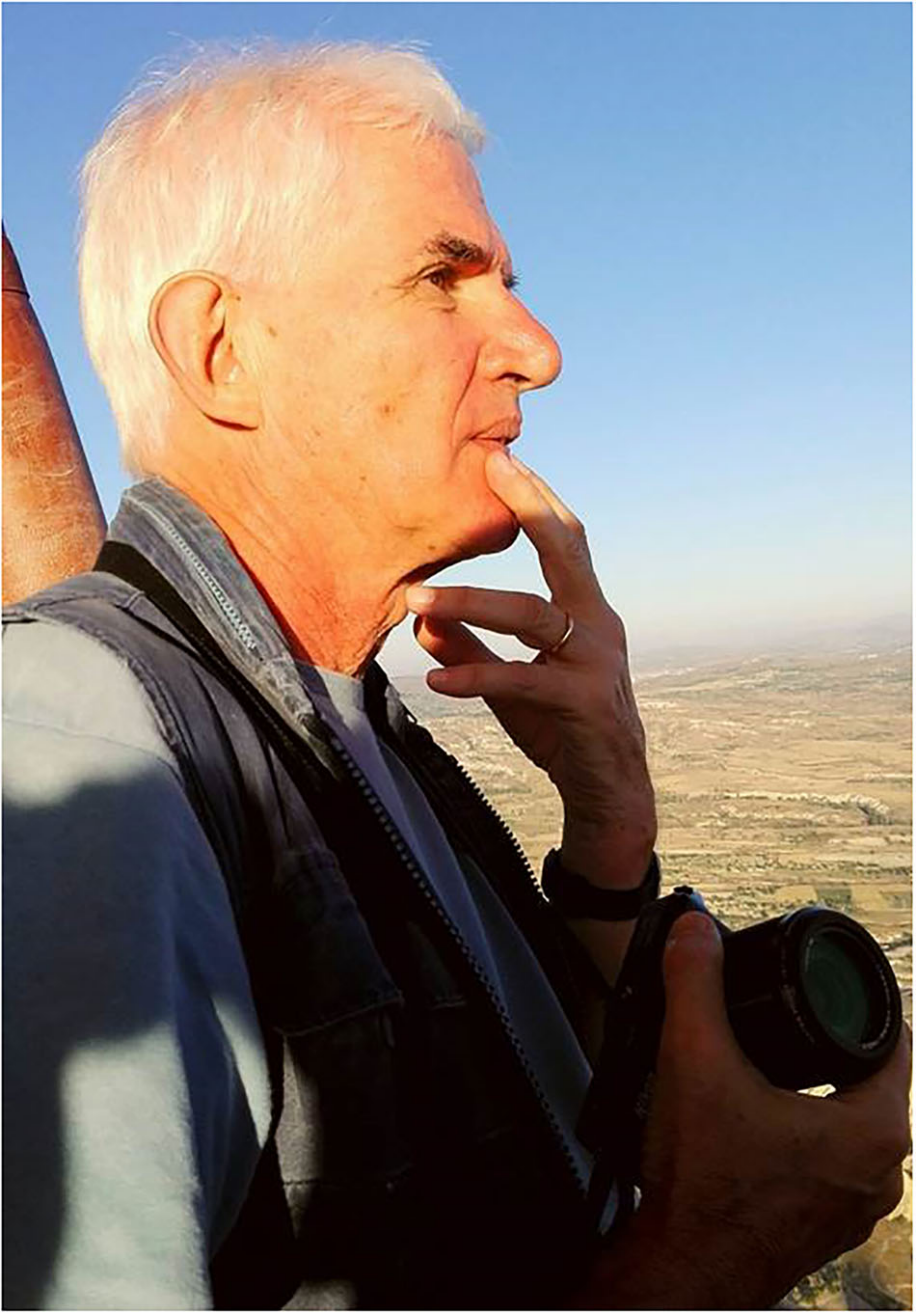
Francesco Di Virgilio (1954–2024). Finis vitae sed opus pergit.

This paper was conceived in Ferrara, in September 2024, during discussions between Francesco di Virgilio (Fig. 1) and Darek Gorecki on shared concerns regarding the methodological inconsistencies and limitations in experimental designs used to demonstrate P2XR activation across various experimental paradigms. Tragically, three weeks later, Francesco died suddenly in Chengdu, where he had participated in the Neuroglia conference. Francesco was deeply respected and loved by the purinergic community, and this paper is a tribute to him and in recognition of his life’s work.

## General considerations

Seven P2X receptor subunits (P2X1-7R) assembled as homomeric and heteromeric receptors are archetypal ligand (ATP)-gated cationic channels permeable to Na^+^, K^+^, and Ca^2+^ involved in numerous physiological and pathological processes. These receptors are widely expressed across various cell types, where they play a crucial role in cell signalling [1]. Activation of P2XRs by extracellular ATP (eATP) contributes to diverse cellular functions, including cell proliferation, differentiation, and death. However, their specific roles in these processes vary significantly depending on the cell type and the particular P2XR subtype(s) involved.

The outcome of P2XR activation is also highly context-dependent and may lead to contrasting effects—for instance, promoting cell death in one cell type while stimulating proliferation and assisting survival in another. This variability underscores the multifaceted P2XR functions but also highlights the potential of these receptors as therapeutic targets in various human diseases. To this end, a variety of selective pharmacological agonists, antagonists, and biologics have been developed, with new therapeutic agents continually emerging [2]. Several of these agents entered clinical trials, emphasising the potential for P2X receptor-targeted therapies [3, 4]. Yet, many trials reported disappointing results [5, 6], which may partly be due to the incomplete understanding of specific functions of P2XRs.

Given the growing momentum in developing therapeutically relevant drugs targeting P2XRs, it seems important to establish a universally accepted set of criteria to validate the specificity and potency of these agents. It is also helpful to provide the scientific community with reliable recommendations detailing assays that can robustly assess P2XR roles across a variety of experimental paradigms. Such standards would allow researchers to better cross-reference findings between laboratories, without stifling the development of new, improved assays or methods. This approach may also provide newcomers to the field a practical toolkit for assessing core P2XR functions.

We present a contemporary set of guidelines developed in consultation with the wide purinergic community. It is intended for researchers to plan and interpret their experiments, and for reviewers to critically assess experimental approaches involving P2XRs. Adherence to these guidelines should enhance the quality of analysis and facilitate the identification of new P2XR functions and therapeutic applications. As experimental methodologies evolve and new assays emerge, these guidelines will need to be revisited and refined. Nevertheless, these will hopefully serve as a foundation for consistent and reliable research practices in P2XR studies.

**Fig. 2:**
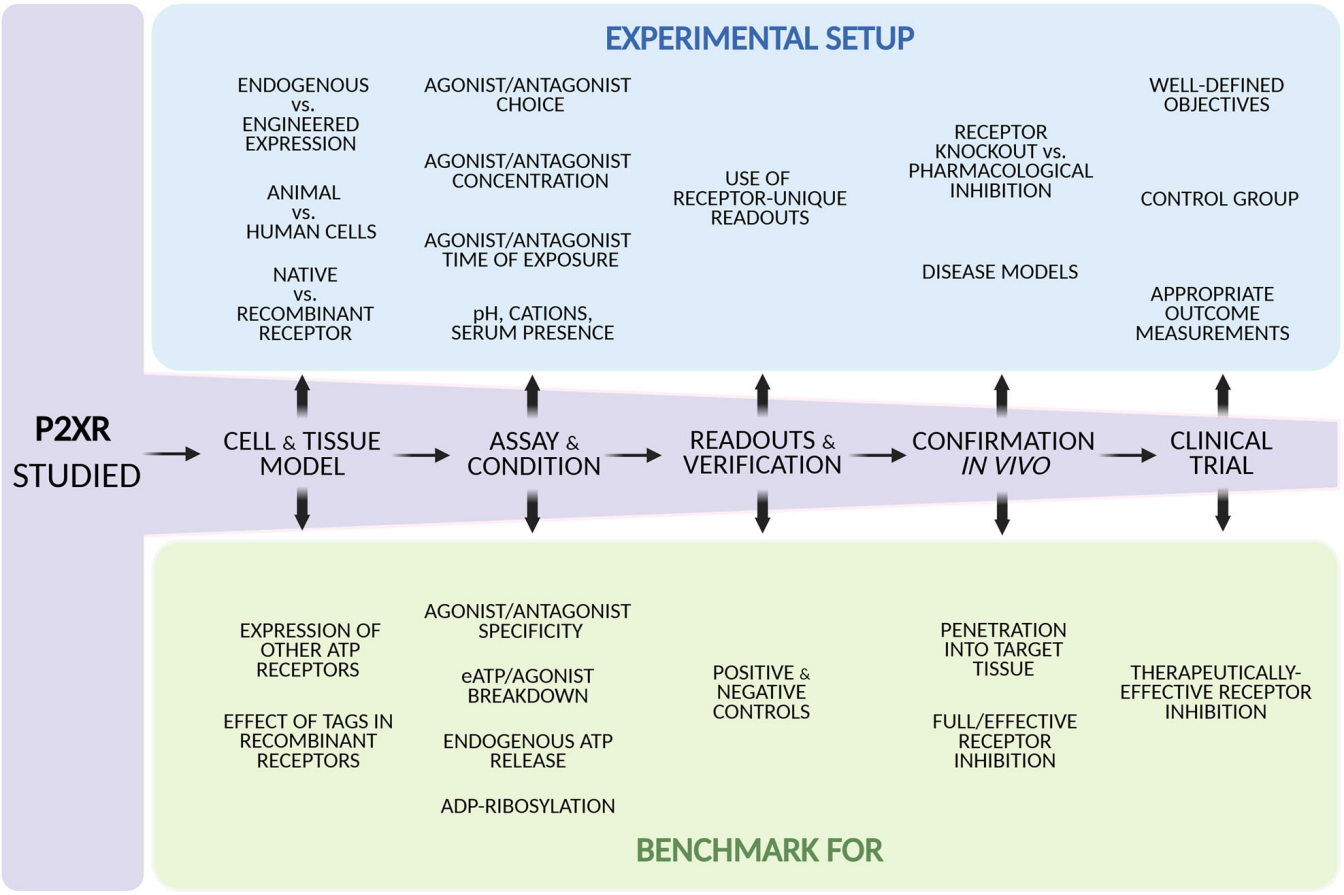
Experimental framework for P2X receptor studies. Flow diagram summarising key considerations for designing and advancing P2XR studies from in vitro to in vivo in health and disease. Experimental design should integrate selection of cell types and expression systems with controls for receptor functionality and subtype specificity. The choice of agonists and antagonists, including their concentration, exposure time, species selectivity, and susceptibility to enzymatic degradation, must be considered. Breakdown of ATP and related agonists generates metabolites (e.g., ADP, adenosine) that activate other receptors. Assay conditions (e.g., temperature, ion composition, pH) can strongly influence P2XR gating and should be standardised. Measurements should use receptor-specific readouts whenever possible, supported by appropriate positive and negative controls to confirm the specificity of observed effects. Novel findings in vitro should ideally be validated in ex vivo systems (e.g., tissue slices, organoids) and in vivo, including in relevant disease models. In vivo experiments must distinguish between a true absence of biological effect and insufficient receptor activation or inhibition by the pharmacological agents used. Identification and application of biomarkers that confirm adequate modulation of receptor activity are critical for interpreting results in preclinical studies and in clinical trials. All these aspects are explored in depth in the subsequent sections.

Analysing P2XR functions requires several key factors to be considered (Fig. 2), as these receptors exhibit significant functional diversity despite shared characteristics. Specifically, although ATP is the primary agonist for P2XRs, different subtypes have varying response thresholds, spanning the nM to mM range. Furthermore, different P2XRs have distinct desensitisation kinetics; kinetics of receptor-generated ion current and ATP stability are temperature-dependent and pH-sensitive. Therefore, the pH and temperature of the assay medium must be controlled, and agonist application timing optimised. The functional inhibition by extracellular divalent cations must also be considered (see section: *The electrophysiological methods in P2X receptor studies*).

The release of endogenous ATP can be triggered, in a cell-specific manner, by relatively trivial manipulations of cells in culture, and affect the experimental outcome. On the other hand, eATP degradation occurring in the course of the experiment can diminish the P2XRs activation but also produce bioactive breakdown products (e.g., adenosine), activating other receptors, often with opposing functions (see section: *Control for ATP release and degradation*).

Additionally, specific assays may not be applicable across all cell types, tissues, or organisms, with some noticeable species-specific P2XR differences. Primary cell cultures (e.g., neuroglia, immune cells) often express multiple P2XR subtypes whose expression can vary with time in culture and culture conditions. Recombinant expression of specific P2XRs provides more controlled conditions, but such systems should be controlled for the effects of receptor overexpression, altered receptor trafficking, and also ensure the appropriate receptor interactome for the physiologically relevant readouts. Moreover, some cell lines can express endogenous receptors.

Use of specific positive and negative controls is an obvious, yet sometimes overlooked, requirement in P2XR assays. The application of appropriate receptor antagonists and corresponding vehicle controls, knockout (KO) cells and organisms, molecular, biochemical, and immunodetection methods confirming the target receptor presence ensures the desired outcome.

The choice of assay/detection methods also requires consideration. Electrophysiology can provide direct ion flux measurements and assess conductance changes, while Ca^2+^ imaging extends the understanding of functional P2XR activation after the opening of the channel (see sections: *The electrophysiological methods in P2X receptor studies and Calcium imaging in P2X receptor studies*). Furthermore, some P2XRs evoke unique functional responses that can be measured (see section: *P2X7 receptor-specific assays*).

Finally, it is important to recognise that a significant portion of data generated in isolated cells maintained in vitro may not accurately reflect the physiological roles of P2XRs in vivo, where the function is often modulated by cell-cell interactions and various physiological and pathological factors. Therefore, in vitro studies must be interpreted with caution and ideally replicated in vivo or in organotypic models to fully understand the functional relevance of P2XR signalling (see sections: *Selection of cellular models and experimental strategies and Assessing P2X receptors* in vivo).

### ATP release as a confounding factor, and quantification of eATP

ATP release occurs through multiple pathways, including exocytosis, membrane channels, and passive leakage from damaged cells. While it serves diverse biological roles and can also contribute to pathological conditions, it also needs to be reckoned with when designing any P2XR experiments.

ATP can be released when the cell membrane potential changes, upon agonist or hormone stimulation, hypoxia, or in response to mechanical disturbances such as shear or osmotic stress. The latter phenomena can occur in physiological situations, such as fluid flow in pre-urine in kidney tubule or secreting or distending epithelium [7–9], but also in experimental contexts during perfusion or injection of drugs into a plate reader, or even with pipetting [8, 10]. Therefore, in a specific cell type studied, control experiments with the application of an isotonic physiological solution without any drugs are needed. In cells with high metabolic activity, nutrient (e.g., glucose) concentration in the medium can quickly lead to elevated eATP due to increased intracellular ATP production and release [11]. Therefore, there is a need to control eATP at the plasma membrane, as this will influence P2XR expression and responses to added ATP analogues and drugs.

As of today, several methods have been developed for eATP quantification. Microelectrode biosensors allow eATP measurements in vivo and provide high accuracy, but require specialised equipment and extensive tissue manipulation, which may cause ATP release [12]. High-performance liquid chromatography (HPLC)-based detection offers precise ATP quantification; at the same time, it requires extensive sample preparation, is unsuitable for in vivo applications, and suffers from low sensitivity, resulting in poor temporal resolution [13].

Fluorescent ATP sensors, such as ATeams, iATPSnFR1.0 [11], ATPOS [14], and GRABATP1.0 [15], utilise fluorescence resonance energy transfer (FRET) or green fluorescent protein (GFP)-based strategies to enable real-time ATP monitoring, including in vivo applications [16, 17]. While fluorescent probes provide high spatial resolution, and visualisation of healthy versus dying cells, highly advanced microscopes and tissue/animal set- ups are required. Tissue penetration by these probes should also be considered [18].

Bioluminescent methods, particularly firefly luciferase-based, remain the most reliable for quantifying eATP [19]. Luciferase-based assays may be “off-line” when ATP is measured in the media isolated from cell cultures or ‘on-line’ when ATP is measured directly in cell cultures or tissues. Online measurements can be performed by the addition of soluble luciferin-luciferase mixtures to media directly above cells to record ATP release following pharmacological or mechanical stimulation [20]. Alternatively, a luciferase enzyme bound to the cell surface with an anchor protein, e.g., *Staphylococcus* protein A for the spaLUC probe can be used [21], with exogenous luciferin substrate added to the system. In an online assay, luminescence can be rapidly triggered, but the readout may linger after ATP has been degraded, thus providing real-time measurements for ATP release but not its removal. Soluble luciferase-based assays are broadly used in vitro and in biofluids collected by microdialysis [22, 23], but not suitable for in vivo applications [19, 24]. The pmeLUC biosensor, developed by Francesco Di Virgilio’s group, overcomes this limitation by anchoring luciferase to the cell membrane [25], thus ensuring precise real-time eATP recording in the immediate cell vicinity and in various disease models, including tumours [26], diabetes [27], inflammation [28], and graft rejection [29]. Additionally, cancer cells can be transfected with the pmeLUC probe and introduced into a host to study eATP dynamics within the tumour microenvironment (TME) [30].

Transgenic pmeLUC mice allow systemic ATP monitoring, aiding studies on ATP-mediated inflammation and neurological disorders [27], and eATP fluctuations in the CNS following cerebral artery occlusion [31, 32]. Continuous advancement of luminescent biosensors may enable the mapping of eATP dynamics throughout the human body. The pmeLUC/nilla recently developed by Di Virgilio and co-workers is a dual-reporter system co-expressing pmeLUC and Renilla luciferases and enables signal normalisation improving accuracy (patent applied for and paper under consideration).

## Tools used to study the P2X receptors

### Agonists and antagonists used in P2X receptor research

**Fig. 3:**
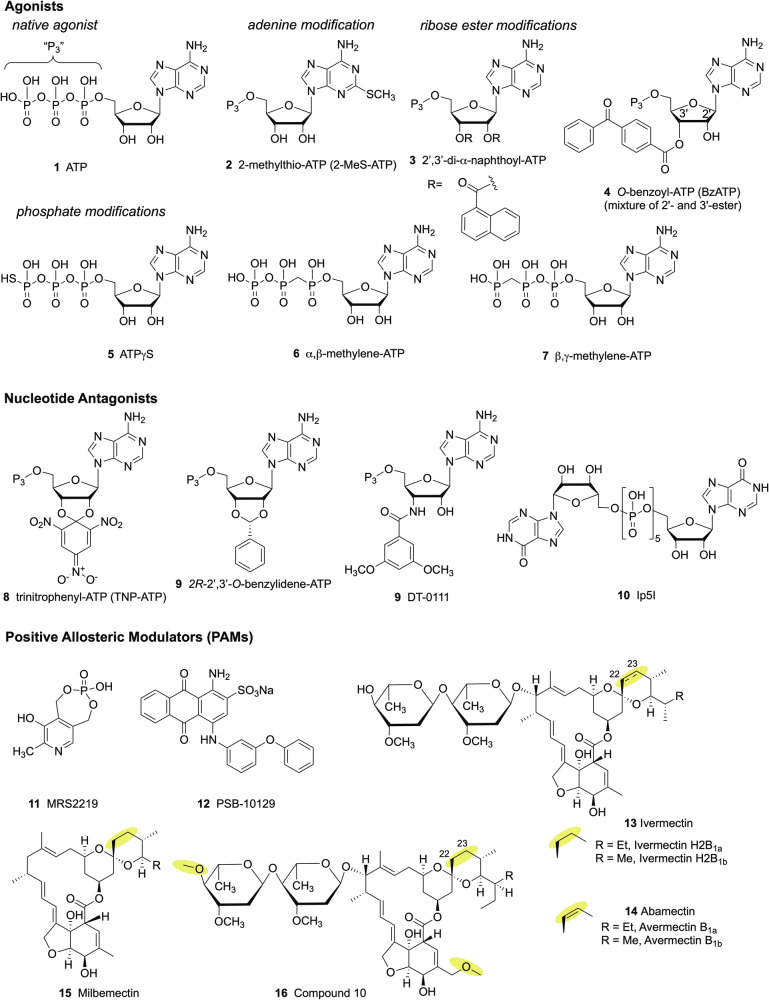
P2XR nucleotide ligands (agonists and antagonists) and PAMs. The yellow highlights on the PAMs indicate key regions of modification. The compound numbers correspond to those listed in bold in the text. Affinities of selected compounds are as cited [34, 37].

**Fig. 4:**
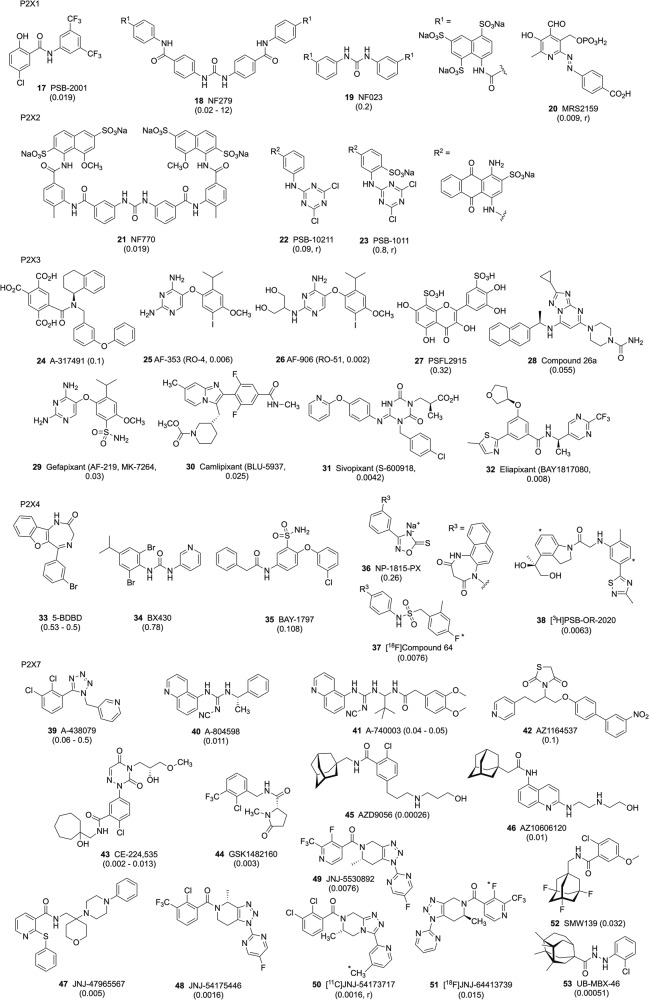
P2XR non-nucleotide antagonists (allosteric and orthosteric). The compound numbers correspond to those listed in bold in the text. The affinities (µM) [33, 35, 38] at the target P2XR (human, unless noted as rat “*r*”) are given in parentheses.

Both nucleotide (Fig. 3) and non-nucleotide antagonists (Fig. 4) for the P2XR have been introduced as research tools [33]. There is a dearth of subtype-selective agonists for use as research tools, but recently, a P2X7R selective antagonist (**[3]**—numbers in square brackets relate to compounds in Figs. 2 and 3) was reported [34]. Many antagonist probes are now a commercially available [35–38].

Exploration of the structure-activity relationships of P2XR ligands has been complicated by the use of non-standard assay methods [36, 39], different pharmacology for hetero- vs. homotrimeric ion channels [33, 40], synthetic challenges [34], the lack (until recently) of structure-based design approaches [38, 40], and the lability of many of the ligands in biological systems [33]. Many of the P2XR antagonists do not compete with ATP, and the elucidation of their allosteric binding enables future ligand discovery [35, 38]. The family of natural products related to ivermectin **[13, 14]** was characterised as P2X4R allosteric enhancers [37]. Their confounding activity at GABA_A_ receptors has been overcome **[16]**, and a simplified scaffold has been shown to retain the P2X4R-enhancing activity **[15]**.

It is important to scrutinise the selectivity and affinity of a given antagonist to a particular species under study, as many display species variability [33, 35, 36], whereas heteromeric P2XRs (e.g., P2X2/3 R) can show unique species differences compared to homomers. Potency shifts mean rodent doses may not predict human therapeutic doses. Moreover, the pharmacokinetics of a given ligand should be considered, as many of these compounds have short half-lives and lack bioavailability, depending on the route of administration. Much effort has been devoted to the introduction of antagonists that cross the blood-brain barrier (BBB), for probing CNS effects [35]. For example, **[44,** **47,** **49, 50**, and **52]** readily cross the BBB. The phosphate-containing ligands, mostly P2XR agonists, cannot penetrate the intact BBB and enter the CNS, and their activity in vivo may reflect their conversion to ADP or adenosine, which activate metabotropic P2Y or adenosine receptors.

Antagonists for P2X3R **[29–32]** and P2X7R have progressed to clinical trials for anti-inflammatory **[43**–**45**, **48**] and psychiatric (JNJ-61393215, JNJ-89495120, not shown) therapeutics [41] and as imaging agents **[44**, **50–52]**, [19, 42]. At least one P2X4R antagonist (NC-2600, structure not disclosed) has progressed to the clinical trial stage for chronic pain [35]. P2X3R antagonists, designed for the treatment of chronic cough, have varying degrees of affinity at heteromeric P2X2/P2X3 receptors, which are responsible for side effects, including reduced taste sensation. Ligands for positron emission tomography (PET) imaging in vivo, containing an ^11^C or ^18^F isotope (mostly for P2X7R, but also for P2X4R), were characterised [35]. Such tools are important for establishing a pharmacokinetics and pharmacodynamics (PK/PD) relationship for experimental drugs, especially for CNS applications. Unfortunately, various P2X7R antagonists have already failed in clinical trials. One possible reason for the failure may be the many naturally occurring single-nucleotide polymorphisms (SNPs) leading to various missense mutations in the P2X7R that are widespread in human populations [42], but other possibilities should also be considered (see chapter: *Assessing p2x receptors* in vivo)

### Modulation of P2X receptors

A key consideration when modulating P2XR activity is the concentration of the agonist. For example, in P2X7R, ATP concentration critically influences the outcome, with low levels promoting pro-survival effects, while high levels can induce cell death [43]. A dose–response experiment is essential to determine the optimal ATP concentration for the desired biological effect and the specific P2XR type.

The duration of agonist exposure is another factor and must be matched to the chosen readout and to the receptor under analysis. Short exposures (seconds) are generally sufficient for assessing ion channel activity, such as Ca^2+^ flux. Longer exposures (minutes) combined with specific readouts identifying the diverse outcomes triggered by different P2XRs (e.g., large pore opening inflammasome activation, proliferation, necroptosis, and receptor shedding for P2X7R [44], can increase the specificity of receptor identification. However, while longer stimulations may be needed, prolonged stimulation can induce cell death, complicating interpretation. Moreover, in the longer-lasting assays, eATP degradation leading to the formation of bioactive metabolites is a significant factor (see section *Control for ATP release and degradation*).

Another important consideration is the timing of antagonist application, whether it should be applied before, concurrently with, or after the agonist, and determining the optimal duration of exposure. The answer to these questions depends on the binding site of the antagonist on the P2XR and whether the presence of the natural ligand, ATP, interferes with the conformation of the binding site [45]. If so, which is the case for many commonly used P2X7R antagonists (AZ10606120, A438079, or A740003), these should be introduced before the agonist, with a pre-treatment period of 30 min to 1 h. If the binding site is unknown, the recommendation would be to test all three modes of stimulation (pre-, co-, and post-application of the ligand).

One should keep in mind that several P2XR agonists, not only ATP but also some used as more subtype-selective, can activate certain P2Y receptors, and these responses should not be confused with P2XR activation.

### Biochemical characterisation of P2X receptors

Recombinant expression of P2XRs using the baculovirus-mediated gene transduction of mammalian cells (BacMam) system can produce enough high-quality receptors for biochemical and structural studies [38, 45–57]. A protocol for expression, detergent solubilisation/reconstitution, and purification of P2XRs is shown as a flowchart in Supplementary Figure 1. P2XRs can be expressed as fusion proteins with an EGFP and octa-histidine affinity tag incorporated at one terminus of each protomer, separated by a protease cleavage sequence to allow removal of both the EGFP and affinity tag before biochemical characterisation of the full trimeric receptor. This approach allows fluorescence size exclusion chromatography (FSEC) to be used for rapid, small-scale ortholog and construct screening as well as identification of optimal purification conditions [57].

The first structural studies of P2XRs, performed with X-ray crystallography on the zebrafish P2X4R (zfP2X4R), the human P2X3R (hP2X3R), the giant panda P2X7R, and the chicken P2X7R, used P2XRs expressed as N-terminal EGFP fusion proteins [45, 48, 49, 51, 58].

**Fig. 5:**
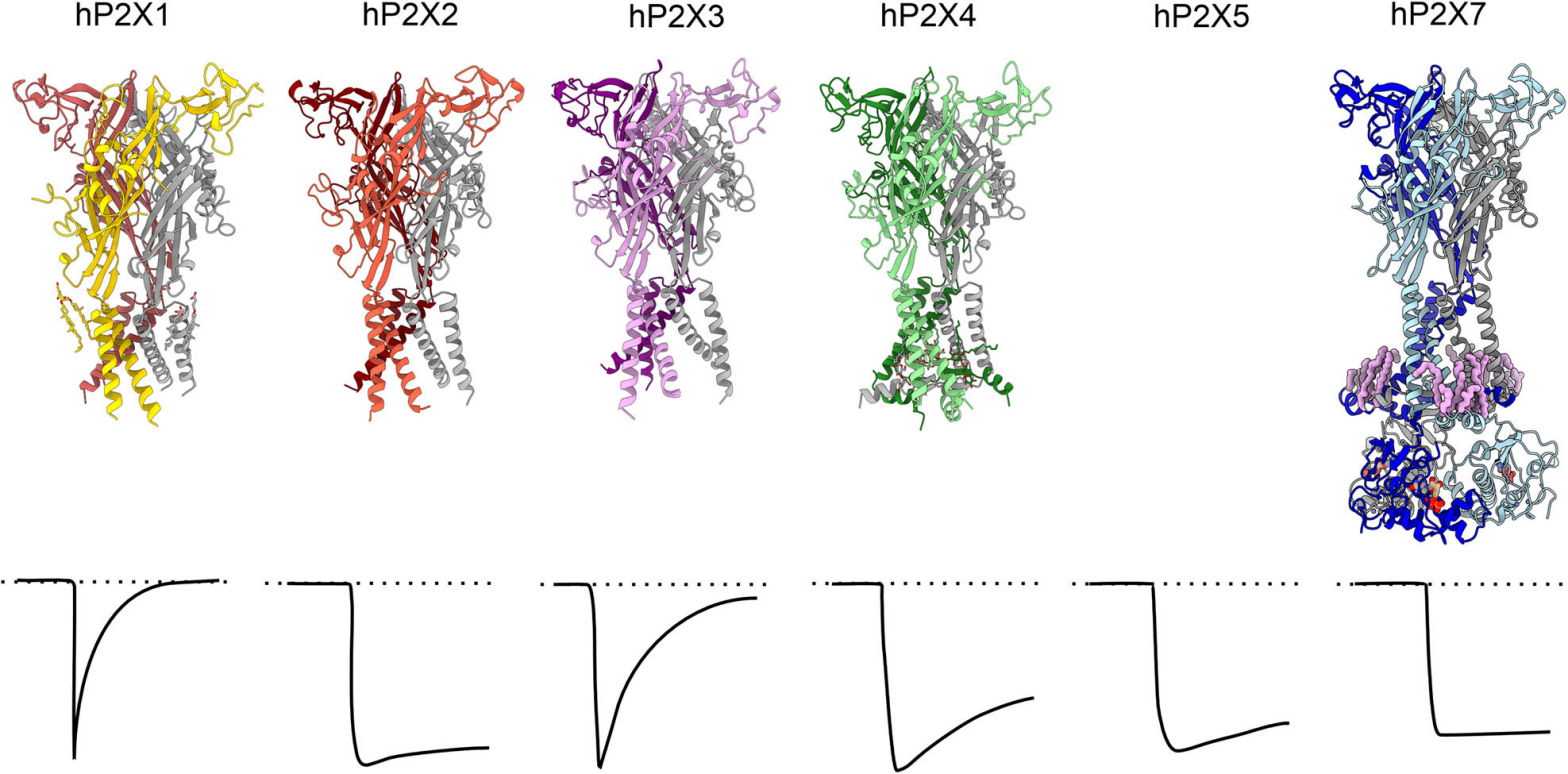
Architecture of P2X receptor subtypes in the apo closed state conformation. Structures in the apo closed state conformation for hP2X1R (PDB: 9C2A) [54], hP2X2R (PDB: 9DDV) [59], hP2X3R (PDB: 5SVJ) [51], hP2X4R (PDB: 9BQH) [56], and hP2X7R (PDB: 9E3M) [38]. For each model, the protomers are coloured differently, with one protomer always shown in grey. The kinetics of currents mediated by homomeric receptors, once the receptor is activated, are shown underneath. The structure of the P2X5R is not available, while P2X6R subunits cannot homo-oligomerise into a functional P2XR [61].

However, in each of these initial expression constructs, the P2XR was truncated to various extents at both the N- and C-termini to facilitate crystallisation. The truncation, in some cases, resulted in receptors with altered gating or even non-functional receptors. Subsequent single particle cryogenic electron microscopy (cryo-EM) studies of full-length wild-type human P2X1R (hP2X1R), human P2X2R (hP2X2R), human P2X4R (hP2X4R), rat P2X7R (rP2X7R), mouse P2X7R, and human P2X7R (hP2X7R) each used a C-terminal fusion protein [38, 52–56, 59]. Dodecyl-β-D-maltopyranoside (C12M), with or without the addition of cholesterol hemi-succinate (CHS), can be utilised for successful detergent reconstitution. The fusion protein is initially isolated with immobilised-metal affinity chromatography (IMAC) and then digested with protease to remove all tags. The final purification step involves separating the trimeric P2XR from the tags and protease with size-exclusion chromatography (SEC) to make the protein suitable for biochemical and/or structural studies (Supplementary Figure 1). The architecture of P2X receptor subtypes in the apo closed state conformation is shown in Fig. 5.

The purification and isolation of P2XRs in an unbound, apo state conformation is complicated by the binding of endogenous ATP released during cell lysis [51, 54, 56, 59]. It is worth noting that this problem is subtype-specific. The P2X7R has the lowest affinity for ATP and does not desensitise; as such, isolating an unliganded, apo state conformation for this subtype was not an issue [38, 52, 53, 55], For each of the desensitising P2XR subtypes, however, standard purification strategies resulted in the isolation of receptors in an ATP-bound, desensitised state conformation, hampering the biochemical and structural analysis of these subtypes in apo closed or ligand-bound inhibited state conformations [46, 51, 54, 56, 59]. To overcome this complication, the purification protocol for the hP2X3R was modified to include extensive dialysis of harvested cell membranes before detergent solubilisation to remove the bound ATP [51]. This protocol was further refined/optimised to isolate the hP2X1R in the apo closed state conformation [54]. This method prevents endogenous ATP from altering conformational states and can be applied to the purification of other P2XR subtypes, such as the hP2X2R [59].

Several ligand-binding assays using purified P2XRs enable the characterisation and quantification of small-molecule/receptor interactions. For example, radioactive filter binding and scintillation proximity assays (SPA) have been used to directly measure the low-nanomolar affinity of radiolabelled ATP (^3^H-ATP) for detergent-solubilized zfP2X4R and hP2X3R, respectively [51, 58]. SPA has also been applied to evaluate competitive antagonist binding to detergent-solubilized hP2X3R by quantifying inhibition or displacement of ^3^H-ATP binding [51]. In contrast, these radioligand-based approaches have been unsuccessful for P2X7R, likely due to its substantially lower affinity for ATP. To address this limitation bio-layer interferometry (BLI) has recently been employed to measure both the binding kinetics and equilibrium affinity of ATP to purified, detergent-solubilized rP2X7R and hP2X7R [38, 53, 55, 60].

These studies provide direct measurements of the dissociation constant (K_D_) and confirm that P2X7R exhibits lower affinity for ATP than other P2XR subtypes [38, 55].

The major advantage of BLI is the possibility of studying the interactions of ligands to receptors without requiring radioactive compounds. While radioactive ATP is readily available and relatively inexpensive, it can only be used to assess the binding of competitive antagonists through displacement assays. The synthesis of radioactive analogues for ligands that bind outside the orthosteric ATP-binding pocket is not easily accomplished. BLI, on the other hand, can be used to evaluate the binding of allosteric antagonists, as was successfully performed for the binding of methyl blue to rP2X7R [53]. It should be noted, though, that measuring the binding of small molecules to the P2X7R is at the edge of detectability for this method (for example, the binding of three ~500 Da molecules of ATP to a P2X7R that is >200 kDa in size), and BLI might not be ideally suited for hydrophobic ligands.

### The electrophysiological methods in P2X receptor studies

Electrophysiological techniques used to study P2XRs, as well as to identify novel subtype-selective agonists and antagonists for clinical applications, include two-electrode voltage-clamp and whole-cell patch-clamp recordings. The pharmaceutical industry leverages high-throughput automated patch-clamp approaches, which enable simultaneous recording from multiple cells [62].

The patch-clamp technique combined with targeted mutagenesis revealed the general characteristics of homo or hetero-trimeric P2XRs (e.g., P2X1/5R, P2X2/3R), the subunit structure–function correlations [63], ATP binding pocket arrangement [64], and agonist/antagonist specificity.

As frog oocytes and HEK293 cells do not express endogenous P2XRs, all subtypes of recombinant P2XRs, and their modifications, can be expressed in these cells following transfection with specific constructs [63]. Patch-clamp recordings demonstrated that the recombinant and native receptors respond identically to ATP. However, it has to be remembered that recombinant receptors expressed in heterologous systems are unlikely to have their native interactome involved in downstream signalling.

Thus, the ion current recordings can reveal molecular events immediately coupled to receptor activation (inward fluxes of Na^+^/Ca^2+^ and outward flux of K^+^). The Ca^2+^ imaging further extends our understanding of P2XRs’ function by supplying information on the secondary increase of [Ca^2+^]_i_ upon opening of the receptor-channel (see section: *Calcium imaging in P2X receptor studies*).

Primary immune cells and either cells isolated from the CNS and peripheral nervous system or brain slices or cells in vivo can be subjected to patch-clamp recordings. The single cells and brain slices can be used acutely or can be kept in culture for a couple of days. Cell culture has the disadvantage that it creates artificial conditions, which may result in a loss or modification of receptor expression.

Construction of agonist concentration–response curves and the study of agonist-antagonist interactions is feasible using submerged organ baths. The bath perfusion has, however, serious drawbacks, because the final drug concentration builds up slowly, and measurements can be made only in the equilibrium state. More advantageous is the pressure application of agonists at single drug concentrations from micropipettes, whereas fast local superfusion systems are optimal, because these allow for the rapid change of minute quantities of drug concentrations around the cell [65, 66]. However, in many cells, fast superfusion or injection can cause ATP release, and this needs to be controlled.

Filling of cells with a fluorescent dye, such as lucifer yellow, helps cell identification. Co-staining using lucifer yellow with post-hoc immunohistochemistry with one of the neuronal, astrocytic, oligodendrocyte, or microglial markers ensures cell-type identification.

It is also worth noting the unique properties of specific P2XRs subtypes, such as rapid desensitisation of P2X1Rs and P2X3Rs [63], agonist sensitivity where P2X7R requires micromolar-millimolar range, considerably higher than P2X1-6 receptors, or the ability to open the large pore on long-lasting or repetitive agonist application (see chapter: *P2X7 RECEPTOR-SPECIFIC ASSAYS*). The functional inhibition by extracellular divalent cations (Ca^2+^, Mg^2+^, Zn^2+^, Cu^2+^) is also a consideration [67], where e.g., P2X7R currents can be amplified by the low divalent cationic conditions in the bath solution [68].

Use of structural analogues of ATP with increased specificity for particular subtypes can help distinguish receptors even further (see section: *Agonists and antagonists used in P2X receptor research*).

### Calcium imaging in P2X receptor studies

Recording changes in intracellular Ca^2+^ concentration ([Ca^2+^]_i_) is used in studies of P2XR function and pharmacology. This method is advantageous for cells growing in suspension or primary cells that are not easily transfected or difficult to patch, such as myeloblasts [69]. Several fluorescent dyes, with specific properties, are being used, including Fura-2 (ratiometric, reduces background noise), Fluo-4 (single-wavelength, easy for high-throughput screening), and GCaMP (genetically encoded Ca^2+^ indicators for cell-specific live imaging). Ratiometric [Ca^2+^]_i_ measurements are based on the monitoring of changes in the Fura-2 emission ratio at 340/380 nm excitation wavelengths, allowing estimation of the actual [Ca^2+^]_i_ [70]. The protocol was developed for cells maintained in suspension, but it can be used for most adherent cell types following their detachment. However, some cells need to adhere to the substrate to show P2XR-dependent Ca^2+^ fluxes. In such cases, it is still possible to use Fura-2 by growing the cells on coverslips at an adequate density, loading them with the dye while still on this support, and mounting the coverslips in a fluorimeter cuvette for measurements [71–73].

Fura-2 signals can be imaged in cells and tissues using microscopes with appropriate light sources (e.g., monochromators, UV lasers) and means of changing fast between the two excitation wavelengths used to monitor calcium signals [74–76]. One of the Fura-2 advantages is that monitoring of individual wavelengths and a ratio can indicate diffusion of the fluorophore out of the cell due to pore formation [77].

When studying responses in cells transfected with a specific P2XR, co-transfection with GFP and Fura-2 imaging allows simultaneous monitoring of adjacent untransfected cells, which do not express the receptor but still take up Fura-2, thereby serving as an internal control [78].

Other techniques are based on live single-cell confocal microscopy with specialised Ca^2+^ sensors. Microplate readers or flow cytometry can be used to assess P2XR-dependent [Ca^2+^]_i_ fluctuations in a large number of cells [70]. Fluo-2 AM, albeit not allowing for quantification of the calcium entry, has been used to detect P2XR-activated [Ca^2+^]_i_ dynamics in primary cells difficult to culture, such as osteoclasts [79] or peripheral neurons [80].

A series of P2X-based eATP sensors was recently developed (see section: *ATP release as a confounding factor, and quantification of eATP*) [81]. However, these sensors depend on cell transfection or genetic engineering of animals for their expression, and therefore, not for measuring naturally occurring P2XR activity.

Several factors must be considered when performing P2XR activity assays with Ca^2+^ sensors. The agonists available are generally not specific for a single subtype. Therefore, to prove the activation of a specific receptor, it is advisable to use controls with selective antagonists whenever available. For P2X4R, for which a selective agonist is not currently available, its effects can be evaluated by potentiating the ATP activity with a selective positive allosteric modulator, such as ivermectin [82, 83] (see section: *Agonists and antagonists used in P2X receptor research*).

Even when proceeding with the transfection of a single subtype in cells not expressing any P2XR, such as the widely used HEK293 cell line, one should keep in mind that the purinergic agonists can activate some P2Y receptors. P2Y are G protein-coupled receptors that trigger Ca^2+^ release from the endoplasmic reticulum as well as store-operated Ca^2+^ entry, thus causing [Ca^2+^]_i_ increases. P2Y-activated Ca^2+^ transients generally have a distinctive shape, as [Ca^2+^]_i_ rises and descends rapidly, so their appearance is different from that of the sustained entry measured for non-desensitising P2XRs. However, to completely exclude a P2Y-dependent component in the [Ca^2+^]_i_ rise activated by the agonist applied, control experiments with no Ca^2+^ in the measurement buffer, and with either EDTA or BAPTA addition to completely chelate extracellular Ca^2+^, can be performed. Under these conditions, any [Ca^2+^]_i_ rise following the administration of an agonist denotes that Ca^2+^ is released from intracellular stores and, therefore, has a P2Y-dependent effect. For more details on the experimental settings, see [43, 84–86].

## P2X7 receptor-specific assays

The P2X7R functions as an ion channel, yet it is distinctive in its low sensitivity to eATP, its near-complete absence of desensitisation [52] and its ability to engage additional signalling and effector mechanisms. Some of these, such as large pore formation, oligomerization domain, leucine-rich repeat receptor, pyrin-domain containing-protein 3 (NLRP3) inflammasome activation, and extracellular vesicle release generally occur upon prolonged stimulation with high eATP concentrations. Low eATP seems to induce signals independent of the channel function [87]. These differences presumably involve the complex interactome (reviewed in ref. [88]), tethered to the unique P2X7R C-terminus. While not yet fully understood, such ion flux-independent responses should be considered when unusual effects of P2X7R activation are identified [89].

### P2X7 receptor and pore formation

One of the differential physiological functions of P2X7R activation when compared to the other P2XRs is the formation of ‘large’ pores (LP) in the plasma membrane. By ‘large’ it is denoted a pore size bigger than the ion channel gate of P2X7R (6.4 Å) up to 900 Da [90].

P2X7R-induced dye uptake has been determined in different cell models and is used as a method to identify P2X7R function. Both the formation and functional significance of the P2X7R LP can be cell-type-specific and also altered in disease states. Cells expressing endogenous P2X7Rs (such as macrophages) or cells expressing it ectopically (HEK293 or oocytes) have all been used to demonstrate P2X7R-induced LP formation [91].

LP formation can be studied employing different plasma membrane-impermeant cationic fluorescent dyes, including Yo-Pro-1 iodide and ethidium bromide. Generally, to stabilise the fluorescent signal, these dyes need to be added 10-15 min before ATP-mediated opening of the P2X7R. Also, use of a physiological buffer [92] or a sucrose-based saline solution [69, 93, 94] rather than cell culture media or PBS is preferable for optimal response, and serum in the assay buffer dramatically reduces the potency of ATP effects (see section: *Impact of serum in the assay buffer*). Dye uptake usually starts 5-10 min after ATP stimulation and lasts for ~30 min [95]. However, in some studies, shorter dye preincubation times were sufficient, and responses were evoked within seconds [96]. Dye uptake can also be assessed in cells in suspension using a fluorimeter with stirred cuvettes, and in slices [97].

Anionic dyes, such as lucifer yellow, can also be used, but seem to present different uptake properties depending on the cell type employed, e.g., being captured by macrophages, but not by HEK293 cells expressing P2X7R [97, 98]. In muscle cells but not in macrophages, autophagy is needed for LP formation and molecules entering through the pore can be targeted to phagophores [99].

Analysis of P2X7R structure, both in apo and ATP-bound states, was unable to identify a receptor state with a dilated pore [52]. Therefore, it is still an open question whether the P2X7R ion channel dilates over time of activation and forms an initial conduit for LP formation that can then be amplified with the activation of accessory proteins. Several proteins have indeed been linked to P2X7R LP formation, including pannexin-1 and gasdermin D. The expression of these proteins will condition the dye uptake found in different cellular models.

When analysing LP formation in lipopolysaccharide (LPS)-primed macrophages, it needs to be considered that P2X7Rs also trigger the NLRP3 inflammasome (see section: *NLRP3 inflammasome activation*); activation of caspase-1 will lead to gasdermin D oligomerization in the plasma membrane and the formation of pores [100]. These pores allow the dyes to pass and could interfere with the identification of P2X7R LP induction. Thus, specific inhibitors of NLRP3 (e.g., MCC950) need to be used to eliminate the contribution of gasdermin D to dye uptake.

### NLRP3 inflammasome activation

When eATP is studied as a pro-inflammatory molecule, classical experimentation relies on the treatment of macrophages with mM concentrations to trigger the P2X7R-induced nucleotide-binding NLRP3 inflammasome. These treatments are usually acute (around 30 min), and the use of a physiological buffer [92] without serum is recommended to obtain the best responses (see section: *Impact of serum in the assay buffer*).

Before application of ATP, macrophages are primed with compounds that activate NF-κB, with various concentrations of bacterial lipopolysaccharide (LPS) being most commonly used, but Pam3CysSerLys4, a synthetic triacylated lipopeptide (Pam3CSK4), is also utilised [101]. The concentrations and time of this stimulation may need optimisation for specific cells and experimental designs. Recombinant cytokines (IFNγ, TNFα) could also be applied, alone or in combination with LPS, but it should be noted that proteins produced in bacterial systems may have LPS contamination.

After priming and before ATP addition, macrophages should be washed thoroughly to remove cytokines produced as a consequence of priming, as well as to remove non-adherent cells. Critical morphological changes can be observed when the P2X7R is triggered for 30 min in primed macrophages, as the plasma membrane of these cells becomes permeable due to pyroptosis, and cells acquire a rounded phenotype with a granular cytoplasm [102, 103].

One of the common measures of the pro-inflammatory actions of eATP in primed macrophages is the release of IL-1ß, since NLRP3 inflammasome induction activates caspase-1, and this enzyme maturates IL-1ß, favouring its release [104]. ELISA for IL-1ß is a fast and quantifiable method, but since IL-1ß is produced as an inactive pro-cytokine during the priming step, an ELISA that mainly recognises mature IL-1ß is highly recommended for the correct interpretation. Alternatively, the proteins in cell supernatants can be concentrated, and detection of mature IL-1ß, active fragments of caspase-1, or Nt-fragment of gasdermin D can be used as markers of inflammasome activation [105].

The outcome of inflammasome activation may vary depending on the cell type. Mouse primary peritoneal macrophages are more responsive to P2X7R activation than mouse bone marrow-derived macrophages, which are widely used in most of the studies [106]. Human primary macrophages derived from monocytes respond to the classical protocol of LPS and ATP stimulation, but due to difficulties in obtaining and variability issues, the human myeloid cell line THP-1 is widely used as a substitute [107]. However, THP-1 requires a differentiation step with phorbol esters (i.e., phorbol 12-myristate 13-acetate, PMA) or IFNγ before the priming step. Various protocols to differentiate THP-1 (in terms of time and concentration of PMA or IFNγ) can produce different, sometimes ill-reproducible results. One verified protocol is described in [108].

For human blood monocytes, ATP stimulation can be done in diluted whole blood or in purified monocytes after LPS priming. Since blood cells are non-adherent, the priming media is not washed, and ATP is added in the presence of LPS stimulation. IL-1ß release needs to be measured by ELISA, as very little of the cytokine is produced. Alternatively, ASC (apoptosis-associated speck-like protein containing a caspase recruitment domain (CARD)) oligomerization can be measured with flow cytometry [109, 110]. This protocol allows for a monocyte-specific assessment of ATP-induced NLRP3 inflammasome formation, and this measure could be used as a standardised method to investigate the immunocompetence of myeloid cells in patients [111].

Additional determinants of inflammatory processes triggered downstream of P2X7R activation in macrophages include inflammatory lipid production (prostaglandin or thromboxane), CD14 shedding, metalloprotease activation, or release of extracellular vesicles [112–114].

### Stimulation of extracellular vesicle release upon P2X7 receptor activation

The release of various types of vesicles from cells mediates the transfer of biomolecules during physiological and pathological processes. The P2X7R-dependent mechanism of extracellular vesicles (EV) release was demonstrated in immune, CNS, and peripheral nervous system [115–117], and in cancer cells [118]. These EVs can carry pro-inflammatory cytokines, miRNA, ATP, and mitochondria [118].

When investigating EVs released upon P2X7R activation, it is important to eliminate external vesicle sources that would interfere with the analysis. Thus, cells to be used in the assay can be grown in a complete culture medium, but since serum-supplemented culture medium contains EVs, once the cells reach the desired density, the medium should be removed, and the cells washed carefully to minimise stress. Cells should be exposed to agonists in either serum-free medium or filtered physiological solutions [23, 116, 119]. P2X7R-dependent vesicular release can be induced either by ATP concentrations ranging from 1 to 3 mM or by BzATP in concentrations between 100 and 300 μM.

Release begins within minutes, and a 15–30 min incubation period is sufficient to collect enough EVs for subsequent characterisation. The cellular debris can be removed from the supernatant with gentle centrifugation, and the vesicles can then be pelleted at high speed, depending on their subtype. The vesicle pellet should be washed to remove residual agonists and re-centrifuged. To preserve vesicle integrity, all steps should be done at 4 °C, and vesicles stored at −80 °C. Repeated freeze-thaw cycles may reduce their quality [120].

Given that P2X7Rs are not the only player involved in the vesicular release process, and that their activation can change their content, the important control step is to collect vesicles from unstimulated cells. These two vesicle populations can be compared in terms of number, size, bioactive content, and biological effects in vitro and in vivo. The characterisation of EVs released upon P2X7R stimulation typically involves confocal and electron microscopy, as well as Nanosight or Videodrop technologies, to assess vesicle size and mitochondrial content. Their cellular origins can be further explored using Western blotting with markers from distinct subcellular compartments [117, 121, 122]. However, this approach remains limited by the current lack of specific markers that reliably distinguish individual EV subclasses or identify vesicles derived from particular subcellular sites. Moreover, the continuous refinement of EV classification and the emergence of newly defined subclasses add a layer of complexity that must be considered [116, 117].

### P2X7 receptor responses, cell adhesion and phagocytosis

During the phagocytosis process, macrophage P2X7Rs are internalised and degraded, and it takes 24–48 h for the cell to replenish its pool at the cell membrane. Moreover, the process of plate adhesion is a form of frustrated phagocytosis. Thus, when conducting P2X7R assays in macrophages, it is advised that any functional analyses, such as permeabilization, Ca^2+^ mobilisation measurements, or IL-1β secretion are performed 24–48 hours after the adhesion step to obtain accurate measurements [123].

Likewise, purinergic signalling analysis during macrophage infection with intracellular parasites requires specific considerations. Some of these infections involve phagocytosis of the parasite. Therefore, any measurements of the infection impact should be performed at least 24 hours after the infection step. However, this is relevant only when infecting macrophages with intracellular bacteria *Chlamydia trachomatis*, or the protozoan *Leishmania amazonensis* [124–126]. This precaution is not necessary when the macrophage infection is caused by the protozoan *Toxoplasma gondii*, since this intracellular parasite actively enters the cells and is not phagocytosed [127].

## Modulators affecting P2X receptor signalling

P2XR function is determined not only by eATP availability but also by eATP flux resulting from both tonic/pulsative release mechanisms and rapid hydrolysis by ectonucleotidases. Function and signalling are also impacted by the surrounding ionic milieu [128, 129], the lipid composition of the membrane [130, 131] and the presence of serum components that can modulate receptor activity.

### Control for ATP release and degradation

**Fig. 6:**
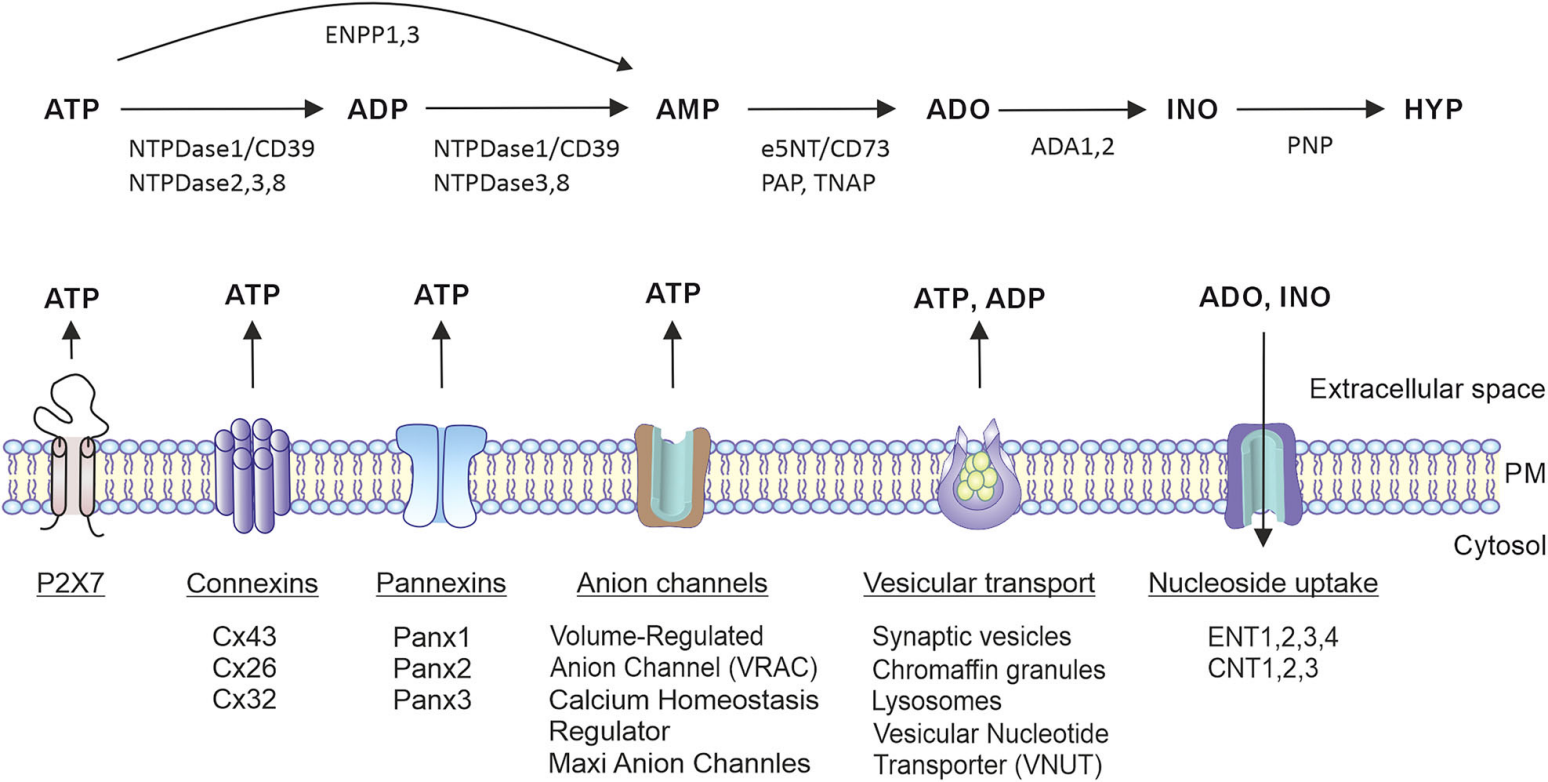
Pathways of cellular ATP release and degradation to other purinergic agonists. Along with massive nucleotide leakage following cell damage, ATP is released through several, often cell-type–specific, non-lytic pathways including vesicular exocytosis, ATP diffusion through plasmalemmal channels such as P2X7R (P2X7), connexin/pannexin hemichannels, and anion channels. Once in the extracellular space, ATP is rapidly degraded. This breakdown shapes the duration and magnitude of ATP signalling and shifts the balance toward its metabolites, which act as agonists at other purinergic receptors. These mechanisms need to be taken into consideration during designing the experiments and data interpretation. The extracellular purine-inactivating pathways include: ecto-nucleoside triphosphate diphosphohydrolase-1 (NTPDase1, also known as CD39), other members of this family, NTPDase2, NTPDase3, and NTPDase8, ecto-nucleotide pyrophosphatase/phosphodiesterase-1 (ENPP1), ecto-5’-nucleotidase (e5NT, also known as CD73), adenosine deaminase (ADA), and purine nucleoside phosphorylase (PNP). For clarity, only the most important purine-inactivating ectoenzymes are shown. Extracellular adenosine (ADO) can be transported into the cells via equilibrative (ENT) or concentrative (CNT) nucleoside transporters, followed by intracellular metabolism. Cx connexin, HYP hypoxanthine, INO inosine, Panx pannexin, PM plasma membrane.

In purinergic signalling, equally important to receptor activation by eATP in an autocrine or paracrine manner are the other components of cellular purine turnover cascade, which include: (i) the release of ATP; (ii) catabolism and inactivation of eATP and other related compounds via a network of purine-converting ectoenzymes; (iii) binding of nucleotide-derived adenosine to G-protein coupled adenosine receptors; and finally, (iv) scavenging of extracellular adenosine by ecto-adenosine deaminase or cellular adenosine uptake through the equilibrative nucleoside transporters (e.g., ENT1/SLC29A1) and concentrative nucleoside transporters (CNTs/SLC28) (Fig. 6). This ATP release and degradation should be taken into consideration when designing the experiments. Specifically, activation of P2X7R and most other P2XRs usually occurs at eATP concentrations within the low millimolar or sub-millimolar range that significantly exceeds the nanomolar ATP levels reported for extracellular fluids and interstitial space [120]. This apparent discrepancy can be explained by focal ATP release and the ability of the cells to retain high concentrations of eATP in the pericellular space without significant nucleotide convection into the bulk milieu [132, 133]. Such relatively high concentrations of eATP at the outer aspect of the plasma membrane are detected in pancreatic beta cells responding to high glucose levels. Simultaneous imaging of eATP and intracellular ATP sensor indicates close coordination between cell metabolism and ATP release [11].

The presence of a complex network of membrane-bound and soluble nucleotide-inactivating and ATP-regenerating enzymes adds another level of complexity to the experimental design, as these regulatory mechanisms govern the duration and magnitude of purinergic responses. Furthermore, cellular adenosine uptake by ENT or CNT, and its intracellular metabolism are expected to have an equally important yet understudied role in activation of adenosine receptor-independent metabolic and epigenetic functions [134–136].

Overall, it is important to consider ATP release and extra- and intra-cellular purine metabolism when studying the biological functions of P2XRs. These are especially relevant, but can be overlooked, under experimental settings of long-term treatment of the cells or tissues with supraphysiological concentrations of exogenous ATP.

### Impact of serum in the assay buffer

Most cell types used to explore purinergic signalling require a complex mix of nutrients, hormones, adhesion molecules, and growth factors in their growth media. These are introduced by the addition of a serum. Foetal bovine serum (FBS) is the most commonly used supplement, human serum is employed where humanised conditions are desired, and sera of other species (e.g., horse) are used for specific applications. Synthetic serum replacements are utilised mostly in cell culture experiments requiring the elimination of animal-derived products [137].

However, the inclusion of serum in the assay may impact results significantly. FBS contains bioactive ATP [22, 138], where the broad range reflects the significant FBS batch-to-batch variability. Thus, cells grown in serum-supplemented media are exposed to varying degrees of purinergic stimulation even before any experimental manipulations. This should be considered in the assay design.

Serum also contains soluble and catalytically active forms of NTPDase1/CD39, ecto-nucleotide pyrophosphatase/phosphodiesterase-1 (ENPP1), adenylate kinase-1, and other purine-converting enzymes [139]. Therefore, additional heat-inactivation of serum samples may be useful for excluding the possibility of undesirable hydrolysis and metabolism of ATP and other purinergic agonists in the assay medium. Furthermore, FBS and horse serum contain active matrix metalloprotease 2 (MMP2) that can cleave the P2X7R on the cell surface. Physiologically, sustained activation of P2X7Rs triggers the release of active MMP2, which, in a feedback loop mechanism cleaves the receptor (amongst other proteins), thus halting its function. This mechanism appears to be universal, operating in cells as diverse as macrophages, dystrophic myoblasts, human tumour cells, and P2X7R-transfected HEK293 cells [140].

Significant MMP2 activity is detected in various sera, and heat inactivation fails to eliminate this activity. As a result, serum concentrations of 5% or higher in the assay buffer have been shown to inhibit P2X7R ion channel function and large pore formation in different cell types [138]. On the other hand, tissue inhibitors of metalloproteinases, especially tissue inhibitor of metalloproteinases 2 (TIMP-2) present in the blood, may inhibit MMP-2 activity, which could explain why P2X7R function can still be measured in whole blood [141].

Consequently, serum should be avoided, or synthetic serum replacements should be considered for specific experiments. Alternatively, MMP2 inhibitors have been shown to effectively restore P2X7R functions. On the other hand, in in vitro studies investigating cells from organs with permeable capillaries, inclusion of blood or serum in the receptor assays may mimic the conditions in vivo.

## Selection of cellular models and experimental strategies

### Cells used in in vitro assays and the ex vivo systems: practical considerations

Native primary cells often express multiple P2XR subtypes, and these can vary under physiological and pathological conditions. This heterogeneity can make the isolation of the contributions of individual P2XR challenging.

Recombinant expression systems, where a target P2XR is overexpressed in a heterologous cell line, allow investigations of precise receptor properties, such as ion permeability and kinetics under well-defined settings [142]. Yet, certain commonly used cell lines can endogenously express some purinergic receptors, especially P2Y receptor subtypes [143] and these background signals can complicate data interpretation.

Therefore, it is essential to verify receptor expression profiles in experimental systems, especially novel ones, through RT-PCR, Western blotting, or functional assays to ensure accurate attribution of responses to the intended P2XR. Moreover, for heterologous models to produce physiologically relevant data, the impact of overexpression, potentially altered receptor trafficking, and the cellular interactome must be carefully considered, as receptor density and the absence of the native membrane composition, intracellular signalling partners, and regulatory proteins can influence receptor functions significantly. Depending on the expression vector used and/or the presence and localisation of a tag (whether N- or C-terminal), or sometimes the location of the mutated site in a recombinant receptor, the expression levels, the localisation, and the functionality of P2XR can be affected. Therefore, it is recommended to characterise the receptor presence, and its correct localisation at the cell membrane before any kind of measurements [144].

This precaution is also mandatory when working with established cell lines that can express multiple variants of P2XRs. Specifically, in the case of P2X7R, specific variants that retain the ion channel activity but lack LP formation have been identified [145, 146]. Among those, a P2X7B splice variant present only in humans has been associated with multiple phenotypes, including cell proliferation [93, 147], cancer progression, metastasis, and therapy resistance [69, 94, 116, 117, 144, 148, 149]. Similarly, the nfP2X7R variant was attributed a role in cancer cell proliferation [146], and clinical trials with an antibody specifically targeting this isoform are ongoing [150, 151]. It is therefore important to characterise all the variants expressed by a cell line, which can be achieved using Western blot analysis probed with antibodies that recognise the receptor variants.

Monocultures of neurons and oligodendrocytes, prepared following standard techniques, are used to evaluate the impact of P2XR agonists and antagonists in e.g., paradigms of stroke [152–155]. While primary cortical neurons and oligodendrocytes are relatively easy to handle in culture, they have limitations when studying purinergic receptors. For example, neurons derived from late embryonic stages require sufficient maturation (at least three weeks in culture) to express P2X7R [152]. In contrast, brain oligodendrocytes are typically obtained through a lengthy procedure (2–3 weeks), often resulting in cultures with some astrocyte contamination. It is also important to consider whether prolonged culture conditions might not artifactually induce the expression of these P2XRs [156]. Alternatively, optic nerve oligodendrocyte cultures mature rapidly (within 4–5 days in vitro) but yield relatively few cells, most of which decline after a week [157].

### P2X receptors in neurogenesis models

P2XRs emerge in early neuronal development and are implicated in neural phenotype determination as well as in neurodegenerative processes [157]. These processes can be modelled using neural differentiation of neural stem cells. Embryos harvested from timed pregnant rats (E12.5–E14.5) or mice (E12.5) can be used for the isolation of embryonic telencephalon [158]. The telencephalons are dissociated, and cells are plated into non-adherent grade dishes for suspension culture and neurosphere formation for 5–10 days in culture medium supplemented with growth factors. Then, neurospheres are collected and replated into laminin/poly-L-lysine-coated dishes for differentiation induction into neurons, astrocytes, and oligodendrocytes. Alternatively, postnatal day 19 (P19) mouse pluripotent cells can be used for neuronal differentiation [158].

The precautions outlined in the sections: *Impact of serum in the assay buffer and P2X7 receptor responses, cell adhesion and phagocytosis*, should be carefully considered.

Differentiated neurospheres should be assayed for neural maturation together with P2XR expression by immunostaining and quantitative image analysis. For that, fixed neurospheres can be immunostained for neuronal β-tubulin, astrocytic glial fibrillary acid protein (GFAP), and oligodendrocyte Gal-C expression [158]. For P2XR immunodetection, the use of primary subunit-selective antibodies of a well-known specificity is important [159]. For quantitative imaging, slides scanned using epifluorescence or confocal microscope can then be subjected to segmentation imaging analysis [160]. 4’,6-Diamidino-2-2phenylindole (DAPI) staining is used for automatic cell nuclei recognition, followed by the analysis of the cells positive for the respective neural antigens and purinergic receptor subtypes. Negative controls in the absence of primary antibodies are needed to determine the threshold. If available, neurospheres from P2XR KO mice can be used to confirm the specificity of the antibody signal.

### A lung tissue slice model for P2X receptor analyses

The precision-cut lung slices (PCLS) can serve as a physiological model that bridges the gap between in vitro cell culture and in vivo animal models [161]. The lung tissue can be collected from mice or humans following lung transplantation surgery. PCLS should retain lung architecture, the extracellular matrix composition, and the differentiated state of all cell populations, including immune cells [162]. A low-melting agarose solution is injected into the trachea, lung inflation is carefully monitored, cold PBS is poured over the lung to solidify the agarose, and then sliced using a vibratome. The sliced lung tissue can be cultured for hours or even a few days in standard culture medium. This approach allows comparing different treatments on P2XRs expression and/or activity, as a single lobe can yield multiple slices, and each slice provides a sufficient number of cells for analysis by flow cytometry, Western blot, or immunohistochemistry. Additionally, this approach enables the use of both healthy and diseased lungs, providing a strong translational link into clinical applications.

## Assessing P2X receptors in vivo

Studying P2XRs in vivo is crucial for bridging the gap between molecular mechanisms and their physiological and pathological effects within the whole organism. Unlike in vitro systems, in vivo studies preserve native cell-cell and cell-extracellular matrix interactions, physiological ion gradients, and the presence or absence of direct contact with serum components (see section: *Impact of serum in the assay buffer*), all of which influence P2X receptor function. The dynamic regulation of ATP release and degradation by ectonucleotidases, which significantly alter receptor activation patterns, can only be accurately assessed in vivo.

**Fig. 7:**
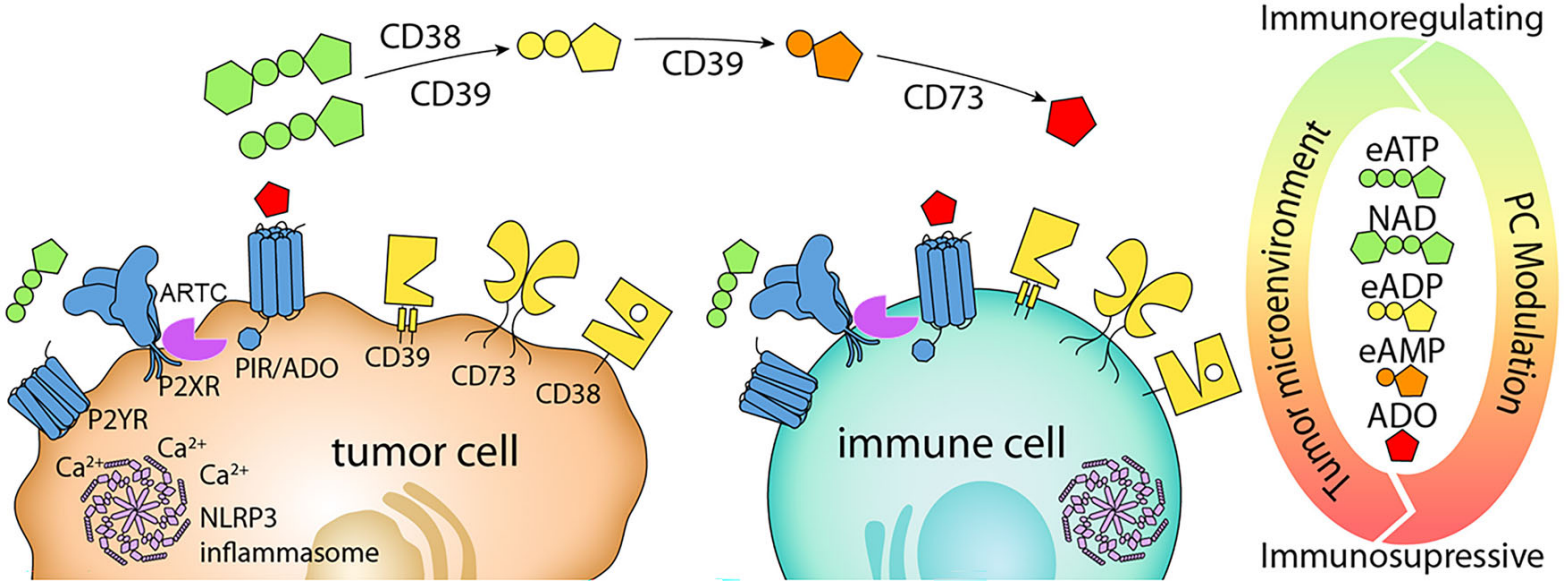
Purinergic signalling in tumours and in antitumor immune responses. The abundance of eATP is a prominent characteristic of the tumour microenvironment (TME). Within the TME, eATP acts either as an immunoregulator or an immunosuppressor, depending on the purinergic components (e.g., receptors and ectonucleotidases) and associated proteins expressed by the tumour and TME cells, and by posttranslational modification, e.g., ADP-ribosylation of P2X7, carried out by species-specific ADP-ribosyltransferases. Abbreviations used: eATP, extracellular ATP. ADO, adenosine, PC, purinergic actors, P2X, purinoreceptor of the P2 family, P1R, purinoreceptor of the P1 family (adenosine receptor), ARTC, ADP-ribosyltransferase, Inflammasome complex (NLRP3, ASC, PYCARD, CASP1). Figure Dr Julia Hambach, University Medical Center Hamburg-Eppendorf, Germany.

For instance, purinergic responses in cancer illustrate the complex interplay between ATP release, specific purinergic receptor activation on various cells, and eATP metabolism with its signalling consequences (Fig. 7). For example, recent studies have demonstrated an interplay between P2X7R, ectonucleotidases and ADORA in cancer progression [117, 163, 164].

Likewise, neuroinflammation involves a complex interplay of P2X7R and P2X4R on microglia and astrocytes, whose functional properties evolve throughout the inflammatory process. However, P2XRs on immune cells also play a role in this response, particularly when these cells gain access to the brain. Similarly, the roles of P2X2R in respiratory regulation and P2X3R in chronic cough can only be fully understood within the physiological context of a living system.

Beyond mechanistic insights, in vivo studies are essential for therapeutic targeting and preclinical validation of novel P2XR agonists and antagonists at physiologically relevant drug concentrations.

### Experimental strategies for investigating P2X receptors in vivo

In vivo studies typically involve pharmacological approaches, genetic models, or a combination of both.

Pharmacological methods utilise P2XR antagonists, which can acutely or chronically block receptor function. JNJ-47965567, a selective P2X7R antagonist, has been shown to reduce mania-like behaviour in the amphetamine-induced hyperactivity [165, 166] and depressive-like behaviour [167]. A-317491, a P2X3R antagonist, effectively reduced chronic and inflammatory pain in rodent models [168, 169], while 5-BDBD, a P2X4R inhibitor, alleviated neuropathic pain [170] and implicated microglia-neuronal signalling in nitroglycerine-induced migraine model [171]. AZ10707120 and A740003 were shown to significantly reduce cancer growth and dissemination in several murine models [116, 172, 173].

Importantly, several considerations such as drug species-specificity [36, 144, 174], hetero-trimer involvement, BBB permeability, off-target effects, and dose selection are crucial when using the pharmacological agents [33].

Drug repurposing approach offers several advantages in drug development, including cost reduction and accelerating treatment availability for various disorders, and is especially important in rare diseases that lack dedicated drug development programmes due to limited commercial incentives. For example, AZT (Zidovudine), a nucleoside reverse transcriptase inhibitor, has been shown to block P2X7R function and alleviate disease progression in the mouse model of Duchenne muscular dystrophy [175].

Genetic approaches include KO models (reviewed in [176]. P2X7R KO mice have been widely used to study neuropathic pain [177], depression [167, 178], neuroinflammation [179], and exocrine gland secretion [180]. Notably, C57BL6 and DBA2 mice commonly used experimentally express an allelic gene variant that results in compromised functionality of the expressed P2X7R, specifically impacting the ability to form pores [181]. Hypofunctional receptor expression can modulate disease phenotypes in mouse models, potentially mitigating or exacerbating observed outcomes.

Several reporter mice have been used to try and identify the cell type-specific expression and localisation of P2X7Rs in the brain. This includes a mouse model which expresses soluble GFP under the mouse *P2rx7* promoter [182], and a reporter mouse with P2X7R C-terminally fused to EGFP [183], Of note, follow-up studies have shown that the reporter mouse expressing soluble GFP also shows increased expression of P2X4Rs and P2X7Rs. Thus, results from this mouse model should be considered with caution [184].

P2X3R KO mice have been used to examine chronic pain [185, 186] and visceral hypersensitivity [187], while P2X4R KO mice help investigate their role in neuropathic pain [170, 188] and affective disorders [189].

### The cell type-specific effects of P2X receptor signalling in vivo—lessons from P2X7R

Different actions involve cell type-specific expression of P2XRs, of which the P2X7Rs have been implicated in numerous seemingly opposing functions, such as pro-inflammatory and pro-apoptotic roles [190–192] vs. promoting cell differentiation, proliferation and cellular survival [84, 193, 194]. While P2X7R expression in immune cells and neuroglia is widely acknowledged, some controversy still exists regarding its presence or absence in neurons [183, 195–197]. In addition to the complex post-transcriptional and post-translational regulation of P2X7Rs [88], different splice variants present different affinities to ATP and downstream signalling profiles [198], possibly adding to cell type-specific differences in P2X7R function. It is therefore important to consider this diversity in experimental paradigms.

P2X7R antagonists reached clinical trials [199, 200], but results were unsatisfactory [41]. Challenges in translating pre-clinical data into the clinic [201–203] may derive from species-specific differences (e.g., rodents vs humans), including possible differences in the cell type-specific expression pattern and downstream signalling landscape. Moreover, when using antagonists in CNS disease models, drug BBB penetration might be an issue, with differential effects in areas with intact and disrupted BBB [201, 202, 204]. Importantly, opposing effects of P2X7R activation on different cell types may dilute results, leading to erroneous/misleading conclusions. For example, while P2X7R deletion in microglia led to a milder seizure and epilepsy phenotype, P2X7R deletion in neurons increased seizure severity [197]. Of note, no effects had been observed in full P2X7R KO mice, with anti- and pro-convulsant functions possibly outweighing each other. Cell type-specific effects may also be the cause of opposing findings using different disease models (e.g., Alzheimer’s disease [205], epilepsy [206] or cancer [201]). Moreover, the impact of P2XR stimulation on certain cell types on other cells (e.g., P2X7R-mediated microglia activation affecting neuronal functioning) should be factored into the experimental design.

Therefore, a cell type-specific deletion of P2XR may provide a more detailed characterisation of the role of this receptor in health and disease. Mouse models, including LoxP mice from the European Conditional Mouse Mutagenesis (EUCOMM) Program and mice expressing floxed humanised P2X7 receptors, are now available [207]. Importantly, dissection of the cell type-specific function of P2XRs at different stages during development or disease progression may allow the development of cell type-specific treatment strategies involving adeno-associated viruses [197, 208] or targeted nanoparticles [209] but also identify ideal treatment windows. While cell-type-specific KO mice are an invaluable tool to establish the cell-type-specific contributions there are certain aspects that need to be taken into consideration. Several Cre lines have been shown to ‘leak’ into other cell types. For example, Cre lines using the *Cx3CR1* promoter to target microglia may also target non-microglial cells such as macrophages, astrocytes, or neurons [210–212]. Cre lines using the *Thy-1* promoter also target T-cells [213], which show a high expression of P2X7Rs [210]. Therefore, expression profiles of Cre lines should be carefully characterised, ensuring P2XR KO is achieved only in the desired cell line. Further, studies using conditional models should ensure that Cre-activating factors, such as tamoxifen [214], do not interfere with the disease model or impact on P2XR expression. P2XR expression in floxed mice may be altered, sometimes in a cell type-specific manner [176]. Thus, careful considerations should be taken when choosing the right controls.

### Behavioural studies to assess P2X receptor functions

The P2XR family plays crucial roles in pain signalling, neuroinflammation, mood regulation, and cognitive processes. Understanding their function in vivo requires well-designed behavioural studies combined with pharmacological approaches and genetically modified animal models.

Behavioural tests should align with the specific P2XR function under investigation. Pain sensitivity can be assessed using von Frey filaments (mechanical pain), the hot plate test (thermal pain), and complete Freund’s adjuvant test (inflammatory pain) [215]. Emotional conditions can be evaluated along the behavioural despair (FST, TST), learned helplessness [216] and anhedonia aspects, open field test (psychomotor agitation (EPM, LDB), and social interaction test (sociability) [217]. Cognitive functions, such as memory and learning, may be examined using the Morris water maze and passive avoidance tests [218]. Exocrine gland function can easily be assessed by the collection of pancreatic juice, saliva, and tears [180].

To ensure robust and reproducible results, baseline behaviour should be recorded before drug administration, as individual variability in locomotion, anxiety, or pain thresholds may markedly influence treatment outcomes. Doses should be selected based on prior pharmacokinetic and pharmacodynamic studies to optimise receptor engagement without inducing off-target effects or toxicity [166, 219, 220]. The timing of behavioural assessments relative to drug exposure should also be carefully standardised, considering both the onset and duration of action. Randomisation, blinding of experimenters, and inclusion of both sexes are essential to minimise bias and increase translational value [221, 222]. Furthermore, detailed reporting of environmental conditions, such as housing, circadian phase of testing, and prior handling, is critical, as P2XR activity can be influenced by stress, immune state, and metabolic factors [223, 224]. Combining behavioural tests with electrophysiology, imaging, and molecular analyses enhances mechanistic understanding and supports the identification of causal links between receptor function and behavioural phenotypes.

### Constraints of experimental disease models in exploring P2X receptor involvement

The roles of P2XRs in stroke and multiple sclerosis have been investigated with intensity. In stroke research, middle cerebral artery occlusion (MCAO) models are widely used to assess their contribution and therapeutic potential ([152] reviewed in ref. [225]). P2X4R and P2X7R have been implicated in reactive microgliosis, neuroinflammation, and ischaemic lesion expansion, whereas P2X1R and P2X3R may contribute to vascular and sensory components of ischaemic brain injury [226, 227]. However, the inherent variability of MCAO models in the extent of ischaemic damage, due to differences in occlusion technique, collateral circulation, and postoperative care, can obscure subtle pharmacological effects. Incomplete representation of human stroke pathophysiology in aged patients or those with comorbidities (e.g., hypertension, diabetes), where P2XR expression and function can shift, is another limitation. Therefore, it is important to standardise the procedure before evaluating the therapeutic potential of subtype-selective ligands.

For multiple sclerosis research, experimental autoimmune encephalomyelitis (EAE) remains a standard immune-driven demyelination model and has been used in demonstrating P2X7R involvement in oligodendrocyte death, axonal damage, and neuroinflammation [152]. However, EAE primarily models T cell-mediated pathology. Complementary non-immune demyelination paradigms, such as cuprizone and lysolecithin exposure, can reveal P2XR-dependent mechanisms intrinsic to oligodendroglia, astrocytes, and microglia (e.g., ATP-driven P2X4R-mediated remyelination, P2X7R-triggered cytotoxicity) [228–231]. Combining these models allows differentiation of immune- versus glia-intrinsic roles of P2XR subtypes, thus improving translational relevance.

Models exploiting specific receptor functionalities should offer a more specific insight into the disease mechanism. For example, a protocol detecting P2X7 receptor activation in vivo in the lungs of mice, based on this receptor’s ability to form a large pore, has been developed. The method involves intranasal administration of TOPRO™-3, a non-permeant DNA-intercalating dye, followed by fluorescence measurement using flow cytometry [232]. eATP enhances TO-PRO™-3 fluorescence mainly in lung immune cells in a P2X7R-dependent manner.

## Receptor modulation and engineering approaches

The low sensitivity of P2X7Rs for ATP can be modulated from the extracellular side of the plasma membrane by other proteins, by posttranslational modification, e.g., ADP-ribosylation of arginine residues, and/or nanobodies and antibodies [233–236].

### Gating P2X7 receptors with ADP-ribosylation of extracellular arginine residues

Gating of P2X7Rs can be modulated by ADP-ribosylation of extracellular arginines [237–239]. This NAD-dependent reaction is catalysed by cell surface ADP-ribosyltransferases ARTC1 and ARTC2 (CD296) (Supplementary Fig. 2) [240–242]. Humans carry a pseudogene for ARTC2, but the orthologue ARTC1 has been reported to ADP-ribosylate P2X7R in human cells [243–245]. The reaction requires co-expression of P2X7Rs and ARTC on the same cell surface, as by mouse immune cells or some tumour cells [245, 246]. For a particular N-terminal variant of mouse P2X7R (P2X7k), ADP-ribosylation at R125 suffices to trigger receptor activation and gating [247]. ADP-ribosylation of other mouse P2X7R variants or human P2X7Rs does not, by itself, gate the receptors, but reduces the threshold for their gating by ATP [240, 248, 249]. Here, ADP-ribosylation resembles the effects of P2X7Rs -potentiating antibodies and nanobodies (see section: *Using antibodies and nanobodies to modulate ATP-mediated gating of P2X7 receptors*).

Notably, the submicromolar quantities of NAD released during routine isolation of primary cells from mouse spleen, liver, or kidney, suffice to induce chronic activation and death of T cell subsets that co-express P2X7k and ARTC2.2, i.e., regulatory T cells (Tregs), tissue resident memory T cells (Trm), and natural killer T (NKT) cells [239, 246, 249, 250].

This process can be controlled by the addition of ARTC2-blocking nanobodies (commercially available) to the medium of a cell culture 10 minutes before harvesting [251]. In mice, injection of the nanobodies intravenously or intraperitoneally [250] or the adeno-associated virus (AAV) vector-driven expression of ARTC2-blocking nanobody in vivo [252] are effective.

### Using antibodies and nanobodies to modulate ATP-mediated gating of P2X7 receptors

A set of P2X7R-specific antibodies and nanobodies from immunised mice, rabbits, and llamas is now available [183, 235, 236, 253–256] (Supplementary Fig. 3). Many of these bind the native receptor without affecting its function. Some, however, effectively potentiate or block the gating of P2X7Rs by ATP [236].

Blocking antibodies inhibit or prevent ATP-induced gating of P2X7Rs. None of the potentiating antibodies or nanobodies selected so far induces gating of P2X7Rs on their own, but they markedly reduce the threshold for gating of P2X7Rs by ATP [236]. Thus, nanobodies and antibodies are useful tools, not only to detect P2X7Rs on cells ex vivo and in vivo, but also to modulate their function in experimental settings [235, 236, 255].

P2X7R-specific nanobodies are stable soluble binding modules that can easily be fused to other nanobodies, the Fc-domain of any antibody isotype, or even the capsid of AAV vectors (Supplementary Fig. 3) [257, 258]. All these constructs can be secreted at high yield by transfected HEK293 cells, and then the product can simply be added to a P2X7R cell assay before or during treatment with ATP.

In mice, nanobodies and heavy chain antibodies can reach P2X7Rs on the surface of parenchymal immune cells in the spleen, liver, and kidney within 10 min after intravenous or intraperitoneal injection [236, 249]. In contrast, targeting P2X7Rs on microglia or immune cells in the brain, requires either stereotactic injection into the cerebrospinal fluid or continued endogenous production in the periphery, e.g., upon intramuscular administration of AAV vectors coding for the selected nanobody [259], as described later.

### Application of AAV vectors coding for selected nanobody-based biologics to modulate P2X7 receptor function in vivo

Based on their properties, nanobodies are a useful novel tool to validate the contribution of P2X7Rs in the pathophysiology of several diseases [31, 239, 255, 259–262]. To avoid repeated nanobody injections, AAV vectors encoding these biologics have been developed to induce their systemic in vivo production (Supplementary Fig. 4). Using this approach, termed AAVnano, a systemic production of anti-P2X7R nanobody-based biologics following a single intramuscular vector-injection can result in serum concentrations ranging from 10 to 40 µg/ml within 14 to 21 days post-transduction, increasing to about 100 µg/ml 30–60 days later [260–262]. Inhibitory or potentiating effects of nanobody-based biologics start to be detectable 7–14 days post AAVnano administration, reaching their maximum effect at around 21 days, and persisting until at least day 120 [252, 260–262].

This AAVnano approach has been used to evaluate the functional contribution of P2X7Rs in several disease animal models, including acute as well as chronic colitis, autoimmune diseases, and several cancer models. The penetrance of the P2X7R blocking nanobody across the blood-brain barrier to reach P2X7R at the surface of microglial cells was evaluated using either direct intracerebroventricular delivery of the recombinant nanobody, its systemic injection, or following the AAVnano strategy, i.e., upon intramuscular administration of AAV vectors coding for the same nanobody-based biologics [259]. Interestingly, 21 days post AAVnano peripheral administration, 80% of the P2X7Rs on the surface of microglial cells were bound with the nanobody-based biologics, which resulted in significant P2X7R inhibition [259].

### Manipulation of P2X receptors by optical control techniques

Given the broad activity of ATP across all P2XRs and most P2Y receptors [263], conventional approaches may lack the specificity to resolve receptor subtype functions in vivo. Emerging optical control strategies, including optopharmacology and optogenetics, enable high-resolution, spatiotemporal modulation of P2XR activity, facilitating precise functional dissection.

Optopharmacology uses light-sensitive molecules or their derivatives to modulate the activity of native receptors. ATP can be linked covalently to a protecting group, such as 4,5-dimethoxy-2-nitrobenzyl (DMNB), 4,5-dimethoxy-2-nitrophenyl (DMNPE), and α-carboxy-2-nitrobenzyl (CNB). These caged compounds remain inactive until exposed to UV light, which releases ATP within milliseconds but in a controlled manner, activating P2XRs in the targeted region [264–267]. A new class of caged ATP derivatives combines photocaging and enhanced lipophilic modifications, enabling more rapid ATP release and passive cell uptake [268]. These compounds are favoured for studying the intracellular P2X pathways, such as that of P2X4 in lysosomes [269] and P2X7 receptors in mitochondria [191].

The concentration of ATP released by a single light pulse can be adjusted to account for the P2XR sensitivity to the agonist (e.g., low for P2X7 receptors). Furthermore, caged receptor-specific or selective agonists or antagonists could be used, as demonstrated for P2Y1/P2Y12 [270] and A_2A_ [271] receptors.

Irreversibility is a drawback of the caged compounds approach [268]. In pursuit of reversible manipulation, photoswitchable ligands are being designed, allowing P2XR channel opening or closing under different wavelengths [272]. Another approach exploited the light- sensitive cis–trans isomerization of the azobenzene, with azobenzene-linked maleimide bound to a cysteine residue introduced into the transmembrane domain, allowing P2X2R, P2X3R, and heteromeric P2X2R/P2X3R receptors channel opening and closing in response to 440 nm or 360 nm wavelengths, respectively [273–275].

This latter approach combines optopharmacological tools with optogenetics, which involves the genetic modification of cells to express proteins whose functions can be controlled by light [276]. Whilst photoswitchable ligands targeting P2XRs have so far been explored in vitro only, optogenetics allows for the precise activation or inhibition of genetically engineered receptors in living tissues [277]. Albeit not yet used for P2XRs, the opsin opto-A_2A_ receptors have demonstrated the potential of this approach in mouse brains [278].

Additionally, chemogenetics that uses engineered receptors designed to respond to specific, novel ligands can be implemented in parallel with optogenetic techniques [279]. A key distinction between optogenetics and chemogenetics is the temporal control over cellular activity. Optogenetics allows for real-time activation or inhibition of P2XRs with millisecond precision, while chemogenetics induces slower responses that last from minutes to hours. Spatial control is also more precise with optogenetics, as light can be delivered to specific regions using fibre-optic cables. In contrast, chemogenetics relies on the viral expression pattern and Cre-driver lines, which may influence the spatial resolution of receptor activation [280].

Optical control tools’ unique challenges include off-target effects and phototoxicity with caged compounds, and compensatory responses and potential functional alterations related to receptor engineering [277, 281]. The selection of optogenetic viral vectors for cell-specific targeting has been covered elsewhere [282], and most studies indicated that at least 3 weeks are necessary to achieve stable viral expression.

## Conclusions

P2X receptor research has advanced significantly in the past two decades, yet specific challenges have delayed translation into actual therapies. We propose that future experimental designs should consider the following challenges, inclusive of: (i) Complex and overlapping receptor expression patterns on multiple cell types making cell populations responsible for a given physiological or pathological effect in vivo difficult to identify unequivocally; (ii) Dynamic regulation and plasticity, where P2XR expression and function changes during physiological process or throughout disease progression; (iii) P2XR forming homo- or hetero-trimers with distinct pharmacology, so results from homomeric studies may not reflect physiological signalling; (iv) A relative paucity of highly selective ligands that can reach the target tissue (e.g. permeate BBB), and the species-specific pharmacology that may contribute to failed translation of animal studies, further exacerbated by animal models and biochemical reactions (e.g., ribosylation) not fully replicating human disease in the context of P2XR regulation; (v) Receptor stability measured in vitro not corresponding with the in vivo conditions, where P2XR activity may be a lot more transient; (vi) eATP breakdown by ectonucleotidases making direct correlation of ATP release with receptor engagement difficult to standardise. These guidelines aim to support experimental design and reliable interpretation of results to improve reproducibility and facilitate translation into clinical applications.

## Supplementary information

Supplementary materials

## References

[1] Verkhratsky A, Burnstock G. Biology of purinergic signalling: its ancient evolutionary roots, its omnipresence and its multiple functional significance. BioEssays [Internet]. 2014;36:697–705. 24782352 10.1002/bies.201400024

[2] Dane C, Stokes L, Jorgensen WT. P2X receptor antagonists and their potential as therapeutics: a patent review (2010-2021). Expert Opin Ther Pat [Internet]. 2022;32:769–90. 35443137 10.1080/13543776.2022.2069010

[3] Birring SS, Cardozo L, Dmochowski R, Dicpinigaitis P, Afzal A, La Rosa C, et al. Efficacy and safety of gefapixant in women with chronic cough and cough-induced stress urinary incontinence: a phase 3b, randomised, multicentre, double-blind, placebo-controlled trial. Lancet Respir Med [Internet]. 2024;12. 10.1016/S2213-2600(24)00222-4 39222649

[4] Yamamoto S, Horita N, Hara J, Sasamoto M, Kanemitsu Y, Hara Y, et al. Benefit-risk profile of P2X3 receptor antagonists for treatment of chronic cough: dose-response model-based network meta-analysis. Chest [Internet]. 2024;166. 10.1016/j.chest.2024.05.015 38857780

[5] Keystone EC, Wang MM, Layton M, Hollis S, McInnes IB. Clinical evaluation of the efficacy of the P2X7 purinergic receptor antagonist AZD9056 on the signs and symptoms of rheumatoid arthritis in patients with active disease despite treatment with methotrexate or sulphasalazine. Ann Rheum Dis. 2012;71:1630–5. 22966146 10.1136/annrheumdis-2011-143578

[6] Stock TC, Bloom BJ, Wei N, Ishaq S, Park W, Wang X, et al. Efficacy and safety of CE-224,535, an antagonist of P2X7 receptor, in treatment of patients with rheumatoid arthritis inadequately controlled by methotrexate. J Rheumatol [Internet]. 2012;39:720–7. 22382341 10.3899/jrheum.110874

[7] Jensen MEJ, Odgaard E, Christensen MH, Praetorius HA, Leipziger J. Flow-induced [Ca2+]i increase depends on nucleotide release and subsequent purinergic signaling in the intact nephron. J Am Soc Nephrol [Internet]. 2007;18:2062–70. 17554149 10.1681/ASN.2006070700

[8] Kowal JM, Yegutkin GG, Novak I. ATP release, generation and hydrolysis in exocrine pancreatic duct cells. Purinergic Signal [Internet]. 2015;11:533–50. 26431833 10.1007/s11302-015-9472-5 PMC4648804

[9] Button B, Okada SF, Frederick CB, Thelin WR, Boucher RC. Mechanosensitive ATP release maintains proper mucus hydration of airways. Sci Signal [Internet]. 2013;6. 10.1126/scisignal.2003755 PMC3791865 23757023

[10] Magni L, Bouazzi R, Olmedilla HH, Petersen PSS, Tozzi M, Novak I. The P2X7 receptor stimulates IL-6 release from pancreatic stellate cells and tocilizumab prevents activation of STAT3 in pancreatic cancer cells. Cells [Internet]. 2021;10:1928. 34440697 10.3390/cells10081928 PMC8391419

[11] Ghiasi SM, Christensen NM, Pedersen PA, Skovhøj EZ, Novak I. Imaging of extracellular and intracellular ATP in pancreatic beta cells reveals correlation between glucose metabolism and purinergic signalling. Cell Signal [Internet]. 2024;117:111109. 38373668 10.1016/j.cellsig.2024.111109

[12] Frenguelli BG, Wall MJ. Combined electrophysiological and biosensor approaches to study purinergic regulation of epileptiform activity in cortical tissue. J Neurosci Methods [Internet]. 2016;260:202–14. 26381061 10.1016/j.jneumeth.2015.09.011

[13] Ledderose C, Valsami EA, Junger WG. Optimized HPLC method to elucidate the complex purinergic signaling dynamics that regulate ATP, ADP, AMP, and adenosine levels in human blood. Purinergic Signal [Internet]. 2022;18:223–39. 35132577 10.1007/s11302-022-09842-w PMC9123122

[14] Imamura H, Huynh Nhat KP, Togawa H, Saito K, Iino R, Kato-Yamada Y, et al. Visualization of ATP levels inside single living cells with fluorescence resonance energy transfer-based genetically encoded indicators. Proc Natl Acad Sci USA [Internet]. 2009;106:15651–6. 19720993 10.1073/pnas.0904764106 PMC2735558

[15] Wu Z, He K, Chen Y, Li H, Pan S, Li B, et al. A sensitive GRAB sensor for detecting extracellular ATP in vitro and in vivo. Neuron [Internet]. 2022;110:770–782.e5. 34942116 10.1016/j.neuron.2021.11.027

[16] Lobas MA, Tao R, Nagai J, Kronschläger MT, Borden PM, Marvin JS, et al. A genetically encoded single-wavelength sensor for imaging cytosolic and cell surface ATP. Nat Commun [Internet]. 2019;10. Available from: https://pubmed.ncbi.nlm.nih.gov/30755613/10.1038/s41467-019-08441-5 PMC6372613 30755613

[17] Kitajima N, Takikawa K, Sekiya H, Satoh K, Asanuma D, Sakamoto H, et al. Real-time in vivo imaging of extracellular ATP in the brain with a hybrid-type fluorescent sensor. Elife [Internet]. 2020;9:1–18. 10.7554/eLife.57544 PMC7398694 32648544

[18] Ozawa T, Yoshimura H, Kim SB. Advances in fluorescence and bioluminescence imaging. Anal Chem [Internet]. 2013;85:590–609. 23134415 10.1021/ac3031724

[19] DeLuca M, McElroy WD. Kinetics of the firefly luciferase catalyzed reactions. Biochem [Internet]. 1974;13:921–5. 10.1021/bi00702a015 4813372

[20] Seminario-Vidal L, Lazarowski ER, Okada SF. Assessment of extracellular ATP concentrations. Methods Mol Biol [Internet]. 2009;574:25–36. 19685297 10.1007/978-1-60327-321-3_3

[21] Okada SF, Nicholas RA, Kreda SM, Lazarowski ER, Boucher RC. Physiological regulation of ATP release at the apical surface of human airway epithelia. Journal of Biological Chemistry [Internet]. 2006;281:22992–3002. Available from: https://www.jbc.org/action/showFullText?pii=S0021925819476382 10.1074/jbc.M603019200 PMC2924190 16754672

[22] Vultaggio-Poma V, Scussel Bergamin L, Falzoni S, Tarantini M, Giuliani AL, Sandonà D, et al. Fetal bovine serum contains biologically available ATP. Purinergic Signal [Internet]. 2024;20:83–9. 37074620 10.1007/s11302-023-09941-2 PMC10828325

[23] Vultaggio-Poma V, Falzoni S, Salvi G, Giuliani AL, Di Virgilio F. Signalling by extracellular nucleotides in health and disease. Biochim Biophys Acta Mol Cell Res [Internet]. 2022. Available from: https://pubmed.ncbi.nlm.nih.gov/35150807/10.1016/j.bbamcr.2022.119237 35150807

[24] Franke H, Grummich B, Härtig W, Grosche J, Regenthal R, Edwards RH, et al. Changes in purinergic signaling after cerebral injury - involvement of glutamatergic mechanisms?. Int J Dev Neurosci [Internet]. 2006;24:123–32. 16387466 10.1016/j.ijdevneu.2005.11.016

[25] Pellegatti P, Falzoni S, Pinton P, Rizzuto R, Di Virgilio F. A novel recombinant plasma membrane-targeted luciferase reveals a new pathway for ATP secretion. Mol Biol Cell [Internet]. 2005;16:3659–65. 15944221 10.1091/mbc.E05-03-0222 PMC1182305

[26] Pellegatti P, Raffaghello L, Bianchi G, Piccardi F, Pistoia V, Di Virgilio F. Increased level of extracellular ATP at tumor sites: in vivo imaging with plasma membrane luciferase. PLoS One [Internet]. 2008. https://pubmed.ncbi.nlm.nih.gov/18612415/10.1371/journal.pone.0002599 PMC2440522 18612415

[27] Tezza, Nasr S, Ben M, D’Addio F, Vergani A, Usuelli V, et al. Islet-derived eATP fuels autoreactive CD8+ T cells and facilitates the onset of type 1 diabetes. Diab [Internet]. 2018;67:2038–53. 10.2337/db17-1227 PMC6905486 30065030

[28] Weber FC, Esser PR, Müller T, Ganesan J, Pellegatti P, Simon MM, et al. Lack of the purinergic receptor P2X(7) results in resistance to contact hypersensitivity. J Exp Med [Internet]. 2010;207:2609–19. 21059855 10.1084/jem.20092489 PMC2989767

[29] Amores-Iniesta J, Barberà-Cremades M, Martínez CM, Pons JA, Revilla-Nuin B, Martínez-Alarcón L, et al. Extracellular ATP activates the NLRP3 inflammasome and is an early danger signal of skin allograft rejection. Cell Rep [Internet]. 2017;21:3414–26. 29262323 10.1016/j.celrep.2017.11.079 PMC5746605

[30] De Marchi E, Orioli E, Pegoraro A, Sangaletti S, Portararo P, Curti A, et al. The P2X7 receptor modulates immune cells infiltration, ectonucleotidases expression and extracellular ATP levels in the tumor microenvironment. Oncogene [Internet]. 2019;38:3636–50. 30655604 10.1038/s41388-019-0684-y PMC6756114

[31] Wilmes M, Pinto Espinoza C, Ludewig P, Stabernack J, Liesz A, Nicke A, et al. Blocking P2X7 by intracerebroventricular injection of P2X7-specific nanobodies reduces stroke lesions. J Neuroinflamm [Internet]. 2022;19:1–14. 10.1186/s12974-022-02601-z PMC9559872 36224611

[32] Faroqi AH, Lim MJ, Kee EC, Lee JH, Burgess JD, Chen R, et al. In vivo detection of extracellular adenosine triphosphate in a mouse model of traumatic brain injury. J Neurotrauma [Internet]. 2021;38:655–64. 32935624 10.1089/neu.2020.7226 PMC7898407

[33] Illes P, Müller CE, Jacobson KA, Grutter T, Nicke A, Fountain SJ, et al. Update of P2X receptor properties and their pharmacology: IUPHAR Review 30. Br J Pharmacol [Internet]. 2021;178. Available from: https://pubmed.ncbi.nlm.nih.gov/33125712/10.1111/bph.15299 PMC8199792 33125712

[34] Isaak A, Dobelmann C, Füsser FT, Erlitz KS, Koch O, Junker A. Unveiling the structure-activity relationships at the orthosteric binding site of P2X ion channels: the route to selectivity. J Med Chem [Internet]. 2022;65:11291–308. 35930402 10.1021/acs.jmedchem.2c00812

[35] Dal Ben D, Buccioni M, Lambertucci C, Francucci B, Smirnov A, Spinaci A, et al. Radioligands targeting the purinergic P2X receptors. Cells [Internet]. 2025;14. Available from: https://pubmed.ncbi.nlm.nih.gov/40643505/10.3390/cells14130984 PMC12248595 40643505

[36] Nagel J, Bous C, Abdelrahman A, Schiedel AC, Müller CE. Species differences of P2X4 receptor modulators. ACS Pharm Transl Sci [Internet]. 2025;8:1320–32. 10.1021/acsptsci.4c00688 PMC12070231 40370986

[37] Meades JL, Bassetto M, Staiculescu MC, Swaminath G, Fountain SJ. Structure–activity relationship of the allosteric effects of ivermectin at human P2X4 and GABAA receptors. Br J Pharmacol [Internet]. 2025; Available from: https://pubmed.ncbi.nlm.nih.gov/40562365/10.1111/bph.70121 40562365

[38] Oken AC, Turcu AL, Tzortzini E, Georgiou K, Nagel J, Westermann FG, et al. A polycyclic scaffold identified by structure-based drug design effectively inhibits the human P2X7 receptor. Nat Commun [Internet]. 2025;16. Available from: https://pubmed.ncbi.nlm.nih.gov/40954149/10.1038/s41467-025-62643-8 PMC12436592 40954149

[39] Dunker C, Vinnenberg L, Isaak A, Karabatak E, Hundehege P, Budde T, et al. Exploring P2X receptor activity: A journey from cellular impact to electrophysiological profiling. Biochem Pharmacol [Internet]. 2024;229. Available from: https://pubmed.ncbi.nlm.nih.gov/39304104/10.1016/j.bcp.2024.116543 39304104

[40] Zuanon M, Brancale A, Young MT. Identification of new human P2X7 antagonists using ligand- and structure-based virtual screening. J Chem Inf Model [Internet]. 2025;65:7143–55. 40566963 10.1021/acs.jcim.5c00552 PMC12264943

[41] Recourt K, de Boer P, van der Ark P, Benes H, van Gerven JMA, Ceusters M, et al. Characterization of the central nervous system penetrant and selective purine P2X7 receptor antagonist JNJ-54175446 in patients with major depressive disorder. Transl Psychiatry [Internet]. 2023;13. Available from: https://pubmed.ncbi.nlm.nih.gov/37482560/10.1038/s41398-023-02557-5 PMC10363543 37482560

[42] Stokes L, Bidula S, Sluyter R. Purinergic P2X receptors as therapeutic targets. Ion Channels as Targets in Drug Discovery [Internet]. 2024; 439–60. Available from: https://link.springer.com/chapter/10.1007/978-3-031-52197-3_13

[43] Adinolfi E, Callegari MG, Ferrari D, Bolognesi C, Minelli M, Wieckowski MR, et al. Basal activation of the P2X7 ATP receptor elevates mitochondrial calcium and potential, increases cellular ATP levels, and promotes serum-independent growth. Mol Biol Cell [Internet]. 2005;16:3260–72. 15901833 10.1091/mbc.E04-11-1025 PMC1165409

[44] Janho dit Hreich S, Benzaquen J, Hofman P, Vouret-Craviari V. To inhibit or to boost the ATP/P2RX7 pathway to fight cancer-that is the question. Purinergic Signal [Internet]. 2021;17:619–31. 34347213 10.1007/s11302-021-09811-9 PMC8677881

[45] Karasawa A, Kawate T. Structural basis for subtype-specific inhibition of the P2X7 receptor. Elife [Internet]. 2016;5:e22153. 27935479 10.7554/eLife.22153 PMC5176352

[46] Bennetts FM, Venugopal H, Glukhova A, Mobbs JI, Ventura S, Thal DM. Structural insights into the human P2X1 receptor and ligand interactions. Nat Commun. 2024;15:1–14 39341830 10.1038/s41467-024-52776-7 PMC11439047

[47] Goehring A, Lee CH, Wang KH, Michel JC, Claxton DP, Baconguis I, et al. Screening and large-scale expression of membrane proteins in mammalian cells for structural studies. Nat Protoc. 2014;9:2574–85. 25299155 10.1038/nprot.2014.173 PMC4291175

[48] Kasuya G, Yamaura T, Ma XB, Nakamura R, Takemoto M, Nagumo H, et al. Structural insights into the competitive inhibition of the ATP-gated P2X receptor channel. Nat Commun [Internet]. 2017;8:1–10. 29026074 10.1038/s41467-017-00887-9 PMC5638823

[49] Kawate T, Michel JC, Birdsong WT, Gouaux E. Crystal structure of the ATP-gated P2X4 ion channel inthe closed state. Nat [Internet. 2009;460:592–8. 10.1038/nature08198 PMC2720809 19641588

[50] Mansoor SE. How structural biology has directly impacted our understanding of P2X receptor function and gating. Methods Mol Biol [Internet]. 2022;2510:1–29. Available from: https://pubmed.ncbi.nlm.nih.gov/35776317/10.1007/978-1-0716-2384-8_1 35776317

[51] Mansoor SE, Lü W, Oosterheert W, Shekhar M, Tajkhorshid E, Gouaux E. X-ray structures define human P2X3 receptor gating cycle and antagonist action. Nature. 2016;538:66–71. 27626375 10.1038/nature19367 PMC5161641

[52] McCarthy AE, Yoshioka C, Mansoor SE. Full-length P2X7 structures reveal how palmitoylation prevents channel desensitization. Cell [Internet]. 2019;179:659–670.e13. 31587896 10.1016/j.cell.2019.09.017 PMC7053488

[53] Oken AC, Ditter IA, Lisi NE, Krishnamurthy I, Godsey MH, Mansoor SE. P2X7 receptors exhibit at least three modes of allosteric antagonism. Sci Adv [Internet]. 2024;10. 10.1126/sciadv.ado5084 PMC11451537 39365862

[54] Oken AC, Lisi NE, Ditter IA, Shi H, Nechiporuk NA, Mansoor SE. Cryo-EM structures of the human P2X1 receptor reveal subtype-specific architecture and antagonism by supramolecular ligand-binding. Nat Commun. 2024;15:1–14. 39353889 10.1038/s41467-024-52636-4 PMC11448502

[55] Oken AC, Lisi NE, Krishnamurthy I, McCarthy AE, Godsey MH, Glasfeld A, et al. High-affinity agonism at the P2X7 receptor is mediated by three residues outside the orthosteric pocket. Nat Commun. 2024;15:1–13. 39107314 10.1038/s41467-024-50771-6 PMC11303814

[56] Shi H, Ditter IA, Oken AC, Mansoor SE. Human P2X4 receptor gating is modulated by a stable cytoplasmic cap and a unique allosteric pocket. Sci Adv. [Internet]. 2025;11. Available from: https://doi.org/10.1126/sciadv.adr3315? 10.1126/sciadv.adr3315 PMC11740937 39823330

[57] Hattori M, Hibbs RE, Gouaux E. A fluorescence-detection size-exclusion chromatography-based thermostability assay for membrane protein precrystallization screening. Struct [Internet]. 2012;20:1293–9. 10.1016/j.str.2012.06.009 PMC3441139 22884106

[58] Hattori M, Gouaux E. Molecular mechanism of ATP binding and ion channel activation in P2X receptors. Nature. 2012;485:7397. 10.1038/nature11010 PMC3391165 22535247

[59] Westermann FG, Oken AC, Granith PKE, Marimuthu P, Müller CE, Mansoor SE. Subtype-specific structural features of the hearing loss-associated human P2X2 receptor. Proc Natl Acad Sci USA [Internet]. 2025;122. Available from: https://pubmed.ncbi.nlm.nih.gov/40938707/10.1073/pnas.2417753122 PMC12452952 40938707

[60] Shah NB, Duncan TM. Bio-layer interferometry for measuring kinetics of protein-protein interactions and allosteric ligand effects. J Vis Exp [Internet]. 2014. Available from: https://pubmed.ncbi.nlm.nih.gov/24638157/10.3791/51383 PMC4089413 24638157

[61] Barrera NP, Ormond SJ, Henderson RM, Murrell-Lagnado RD, Edwardson JM. Atomic force microscopy imaging demonstrates that P2X2 receptors are trimers but that P2X6 receptor subunits do not oligomerize. J Biol Chem [Internet]. 2005;280:10759–65. 15657042 10.1074/jbc.M412265200

[62] Zimmermann D, Kress M, Zeidler M. Biophysical essentials—a full-stack open-source software framework for conserved and advanced analysis of patch-clamp recordings. Comput Methods Programs Biomed [Internet]. 2024;255. Available from: https://pubmed.ncbi.nlm.nih.gov/39038390/10.1016/j.cmpb.2024.108328 39038390

[63] North RA. Molecular physiology of P2X receptors. Physiol Rev [Internet]. 2002;82:1013–67. 12270951 10.1152/physrev.00015.2002

[64] Kowalski M, Hausmann R, Schmid J, Dopychai A, Stephan G, Tang Y, et al. Flexible subunit stoichiometry of functional human P2X2/3 heteromeric receptors. Neuropharmacol [Internet]. 2015;99:115–30. 10.1016/j.neuropharm.2015.07.008 26184350

[65] Oliveira JF, Riedel T, Leichsenring A, Heine C, Franke H, Krügel U, et al. Rodent cortical astroglia express in situ functional P2X7 receptors sensing pathologically high ATP concentrations. Cereb Cortex [Internet]. 2011;21:806–20. 20739479 10.1093/cercor/bhq154

[66] Nörenberg W, Schunk J, Fischer W, Sobottka H, Riedel T, Oliveira J, et al. Electrophysiological classification of P2X7 receptors in rat cultured neocortical astroglia. Br J Pharm [Internet]. 2010;160:1941–52. 10.1111/j.1476-5381.2010.00736.x PMC2958640 20649592

[67] Jiang LH. Inhibition of P2X7 receptors by divalent cations: Old action and new insight. Eur Biophys J [Internet]. 2009;38:339–46. 18414844 10.1007/s00249-008-0315-y

[68] Ren WJ, Zhao YF, Li J, Rubini P, Yuan ZQ, Tang Y, et al. P2X7 receptor-mediated depression-like reactions arising in the mouse medial prefrontal cortex. Cereb Cortex [Internet]. 2023;33:8858–75. 37183178 10.1093/cercor/bhad166

[69] Pegoraro A, Orioli E, De Marchi E, Salvestrini V, Milani A, Di Virgilio F, et al. Differential sensitivity of acute myeloid leukemia cells to daunorubicin depends on P2X7A versus P2X7B receptor expression. Cell Death Dis [Internet]. 2020;11. Available from: https://pubmed.ncbi.nlm.nih.gov/33071281/10.1038/s41419-020-03058-9 PMC7569086 33071281

[70] Di Virgilio F, Jiang LH, Roger S, Falzoni S, Sarti AC, Vultaggio-Poma V, et al. Structure, function and techniques of investigation of the P2X7 receptor (P2X7R) in mammalian cells. Methods Enzymol [Internet]. 2019;629:115–50. 31727237 10.1016/bs.mie.2019.07.043

[71] Solini A, Chiozzi P, Morelli A, Adinolfi E, Rizzo R, Baricordi OR, et al. Enhanced P2X7 activity in human fibroblasts from diabetic patients: a possible pathogenetic mechanism for vascular damage in diabetes. Arterioscler Thromb Vasc Biol [Internet]. 2004;24:1240–5. 15155383 10.1161/01.ATV.0000133193.11078.c0

[72] Yeung D, Zabłocki K, Lien C, Jiang T, Arkle S, Brutkowski W, et al. Increased susceptibility to ATP via alteration of P2X receptor function in dystrophic mdx mouse muscle cells. FASEB J [Internet]. 2006;20:610–20. 16581969 10.1096/fj.05-4022com

[73] Pegoraro A, Bortolotti D, Marci R, Caselli E, Falzoni S, De Marchi E, et al. The P2X7 receptor 489C>T gain of function polymorphism favors HHV-6A infection and associates with female idiopathic infertility. Front Pharm [Internet]. 2020;11:508596. 10.3389/fphar.2020.00096 PMC7046806 32153407

[74] Hede SE, Amstrup J, Christoffersen BC, Novak I. Purinoceptors evoke different electrophysiological responses in pancreatic ducts. P2Y inhibits K+ conductance, and P2X stimulates cation conductance. J Biol Chem [Internet]. 1999;274:31784–91. 10542200 10.1074/jbc.274.45.31784

[75] Haanes KA, Schwab A, Novak I. The P2X7 receptor supports both life and death in fibrogenic pancreatic stellate cells. PLoS One [Internet]. 2012;7:e51164. 23284663 10.1371/journal.pone.0051164 PMC3524122

[76] Tozzi M, Larsen AT, Lange SC, Giannuzzo A, Andersen MN, Novak I. The P2X7 receptor and pannexin-1 are involved in glucose-induced autocrine regulation in β-cells. Sci Rep. 2018;8:1–15. 29895988 10.1038/s41598-018-27281-9 PMC5997690

[77] Tozzi M, Hansen JB, Novak I. Pannexin-1 mediated ATP release in adipocytes is sensitive to glucose and insulin and modulates lipolysis and macrophage migration. Acta Physiol [Internet]. 2020;228:e13360. 10.1111/apha.13360. 10.1111/apha.13360 31400255

[78] Magni L, Yu H, Christensen NM, Poulsen MH, Frueh A, Deshar G, et al. Human P2X7 receptor variants Gly150Arg and Arg276His polymorphisms have differential effects on risk association and cellular functions in pancreatic cancer. Cancer Cell Int [Internet]. 2024;24:1–16. 38664691 10.1186/s12935-024-03339-9 PMC11044319

[79] Orriss IR, Knight GE, Ranasinghe S, Burnstock G, Arnett TR. Osteoblast responses to nucleotides increase during differentiation. Bone [Internet. 2006;39:300–9. 16616882 10.1016/j.bone.2006.02.063

[80] Long J, Lei X, Chen M, Yang S, Sun T, Zeng J, et al. CB1 Receptors Mediated Inhibition of ATP-Induced [Ca2+]i Increase in Cultured Rat Spinal Dorsal Horn Neurons. Neurochem Res [Internet]. 2018;43:267–75. 29127599 10.1007/s11064-017-2414-6

[81] Ollivier M, Beudez J, Linck N, Grutter T, Compan V, Rassendren F. P2X-GCaMPs as Versatile Tools for Imaging Extracellular ATP Signaling. eNeuro [Internet. 2021;8:1–15. 10.1523/ENEURO.0185-20.2020 PMC7877454 33380526

[82] Seil M, El Ouaaliti M, Fontanils U, Etxebarria IG, Pochet S, Dal Moro G, et al. Ivermectin-dependent release of IL-1beta in response to ATP by peritoneal macrophages from P2X(7)-KO mice. Purinergic Signal [Internet]. 2010;6:405–16. 21437011 10.1007/s11302-010-9205-8 PMC3033504

[83] Sluyter R, McEwan TBD, Sophocleous RA, Stokes L. Methods for studying P2X4 receptor ion channels in immune cells. J Immunol Methods [Internet]. 2024;526. Available from: https://pubmed.ncbi.nlm.nih.gov/38311008/10.1016/j.jim.2024.113626 38311008

[84] Adinolfi E, Callegari MG, Cirillo M, Pinton P, Giorgio C, Cavagna D, et al. Expression of the P2X7 receptor increases the Ca2+ content of the endoplasmic reticulum, activates NFATc1, and protects from apoptosis. Journal of Biological Chemistry [Internet]. 2009;284:10120–8. Available from: https://www.jbc.org/action/showFullText?pii=S0021925820322742 10.1074/jbc.M805805200 PMC2665066 19204004

[85] Pastore S, Mascia F, Gulinelli S, Forchap S, Dattilo C, Adinolfi E, et al. Stimulation of purinergic receptors modulates chemokine expression in human keratinocytes. J Invest Dermatol [Internet]. 2007;127:660–7. 17039239 10.1038/sj.jid.5700591

[86] Rossi L, Manfredini R, Bertolini F, Ferrari D, Fogli M, Zini R, et al. The extracellular nucleotide UTP is a potent inducer of hematopoietic stem cell migration. Blood [Internet]. 2007;109:533–42. 17008551 10.1182/blood-2006-01-035634

[87] Amstrup J, Novak I. P2X7 receptor activates extracellular signal-regulated kinases ERK1 and ERK2 independently of Ca2+ influx. Biochem J [Internet]. 2003;374:51–61. 12747800 10.1042/BJ20030585 PMC1223572

[88] Kopp R, Krautloher A, Ramírez-Fernández A, Nicke A. P2X7 interactions and signaling—making head or tail of it. Front Mol Neurosci [Internet]. 2019;12. Available from: https://pubmed.ncbi.nlm.nih.gov/31440138/10.3389/fnmol.2019.00183 PMC6693442 31440138

[89] Carrasquero LMG, Delicado EG, Sánchez-Ruiloba L, Iglesias T, Miras-Portugal MT. Mechanisms of protein kinase D activation in response to P2Y2 and P2X7 receptors in primary astrocytes. Glia [Internet]. 2010;58:984–95. 20222145 10.1002/glia.20980

[90] Wei L, Caseley E, Li D, Jiang LH. ATP-induced P2X receptor-dependent large pore formation: how much do we know? Front Pharmacol [Internet]. 2016;7. Available from: https://pubmed.ncbi.nlm.nih.gov/26858647/10.3389/fphar.2016.00005 PMC4732382 26858647

[91] Pelegrin P, Surprenant A. The P2X(7) receptor-pannexin connection to dye uptake and IL-1beta release. Purinergic Signal [Internet]. 2009;5:129–37. 19212823 10.1007/s11302-009-9141-7 PMC2686830

[92] Barberà-Cremades M, Gómez AI, Baroja-Mazo A, Martínez-Alarcón L, Martínez CM, de Torre-Minguela C, et al. P2X7 receptor induces tumor necrosis factor-α converting enzyme activation and release to boost TNF-α production. Front Immunol. 2017;8:250503. 10.3389/fimmu.2017.00862 PMC5523084 28791020

[93] Adinolfi E, Cirillo M, Woltersdorf R, Falzoni S, Chiozzi P, Pellegatti P, et al. Trophic activity of a naturally occurring truncated isoform of the P2X7 receptor. FASEB J [Internet]. 2010;24:3393–404. 20453110 10.1096/fj.09-153601

[94] Zanoni M, Sarti AC, Zamagni A, Cortesi M, Pignatta S, Arienti C, et al. Irradiation causes senescence, ATP release, and P2X7 receptor isoform switch in glioblastoma. Cell Death Dis [Internet]. 2022;13. Available from: https://pubmed.ncbi.nlm.nih.gov/35075119/10.1038/s41419-022-04526-0 PMC8786947 35075119

[95] Pelegrin P, Surprenant A. Pannexin-1 mediates large pore formation and interleukin-1β release by the ATP-gated P2X7 receptor. EMBO J [Internet]. 2006;25:5071–82. 17036048 10.1038/sj.emboj.7601378 PMC1630421

[96] Jursik C, Sluyter R, Georgiou JG, Fuller SJ, Wiley JS, Gu BJ. A quantitative method for routine measurement of cell surface P2X7 receptor function in leucocyte subsets by two-colour time-resolved flow cytometry. J Immunol Methods [Internet]. 2007;325:67–77. 17618646 10.1016/j.jim.2007.06.002

[97] Falzoni S, Munerati M, Ferrari D, Spisani S, Moretti S, Di Virgilio F. The purinergic P2Z receptor of human macrophage cells: characterization and possible physiological role. J Clin Investig [Internet]. 1995;95:1207–16. 7883969 10.1172/JCI117770 PMC441459

[98] Schachter J, Motta AP, Zamorano A, de S, da Silva-Souza HA, Guimarães MZP, et al. ATP-induced P2X7-associated uptake of large molecules involves distinct mechanisms for cations and anions in macrophages. J Cell Sci [Internet]. 2008;121:3261–70. 18782864 10.1242/jcs.029991

[99] Young CNJ, Sinadinos A, Lefebvre A, Chan P, Arkle S, Vaudry D, et al. A novel mechanism of autophagic cell death in dystrophic muscle regulated by P2RX7 receptor large-pore formation and HSP90. Autophagy [Internet]. 2015;11:113–30. 25700737 10.4161/15548627.2014.994402 PMC4502824

[100] Broz P, Pelegrín P, Shao F. The gasdermins, a protein family executing cell death and inflammation. Nat Rev Immunol [Internet]. 2020;20:143–57. 31690840 10.1038/s41577-019-0228-2

[101] Fernandes-Alnemri T, Kang S, Anderson C, Sagara J, Fitzgerald KA, Alnemri ES. Cutting edge: TLR signaling licenses IRAK1 for rapid activation of the NLRP3 inflammasome. J Immunol [Internet]. 2013;191:3995–9. 10.4049/jimmunol.1301681. 24043892 10.4049/jimmunol.1301681 PMC3924784

[102] Virginio C, Mackenzie A, North RA, Surprenant A. Kinetics of cell lysis, dye uptake and permeability changes in cells expressing the rat P2X7 receptor. J Physiol [Internet]. 1999;519:335–46. 10457053 10.1111/j.1469-7793.1999.0335m.x PMC2269518

[103] Mackenzie AB, Young MT, Adinolfi E, Surprenant A. Pseudoapoptosis induced by brief activation of ATP-gated P2X7 receptors. J Biol Chem [Internet]. 2005;280:33968–76. 15994333 10.1074/jbc.M502705200

[104] Pelegrin P. P2X7 receptor and the NLRP3 inflammasome: partners in crime. 2021;187:114385. Available from: 10.1016/j.bcp.2020.114385 10.1016/j.bcp.2020.114385 33359010

[105] Molina-López C, Hurtado-Navarro L, García CJ, Angosto-Bazarra D, Vallejo F, Tapia-Abellán A, et al. Pathogenic NLRP3 mutants form constitutively active inflammasomes resulting in immune-metabolic limitation of IL-1β production. Nat Commun [Internet]. 2024;15. Available from: https://pubmed.ncbi.nlm.nih.gov/38321014/10.1038/s41467-024-44990-0 PMC10847128 38321014

[106] Pelegrin P, Barroso-Gutierrez C, Surprenant A. P2X7 receptor differentially couples to distinct release pathways for IL-1beta in mouse macrophage. J Immunol [Internet]. 2008;180:7147–57. 18490713 10.4049/jimmunol.180.11.7147

[107] Pelegrin P, Surprenant A. Pannexin-1 mediates large pore formation and interleukin-1beta release by the ATP-gated P2X7 receptor. EMBO J [Internet]. 2006;25:5071–82. 17036048 10.1038/sj.emboj.7601378 PMC1630421

[108] Baroja-Mazo A, Compan V, Martín-Sánchez F, Tapia-Abellán A, Couillin I, Pelegrín P. Early endosome autoantigen 1 regulates IL-1β release upon caspase-1 activation independently of gasdermin D membrane permeabilization. Sci Rep. 2019;9:1–10. 30962463 10.1038/s41598-019-42298-4 PMC6453936

[109] Hurtado-Navarro L, Baroja-Mazo A, Pelegrín P. Characterization of P2X7 Receptors in Human Blood Cells. Methods Mol Biol [Internet]. 2022;2510:279–90. 35776331 10.1007/978-1-0716-2384-8_15

[110] Hurtado-Navarro L, Baroja-Mazo A, Martínez-Banaclocha H, Pelegrín P. Assessment of ASC oligomerization by flow cytometry. Methods Mol Biol [Internet]. 2022;2459:1–9. 35212949 10.1007/978-1-0716-2144-8_1

[111] Martínez-García JJ, Martínez-Banaclocha H, Angosto-Bazarra D, de Torre-Minguela C, Baroja-Mazo A, Alarcón-Vila C, et al. P2X7 receptor induces mitochondrial failure in monocytes and compromises NLRP3 inflammasome activation during sepsis. Nat Commun [Internet]. 2019;10. Available from: https://pubmed.ncbi.nlm.nih.gov/31221993/10.1038/s41467-019-10626-x PMC6586640 31221993

[112] Barberà-Cremades M, Baroja-Mazo A, Gomez AI, Machado F, Di Virgilio F, Pelegrín P. P2X7 receptor-stimulation causes fever via PGE2 and IL-1β release. FASEB J [Internet]. 2012;26:2951–62. 10.1096/fj.12-205765. 22490780 10.1096/fj.12-205765

[113] De Torre-Minguela C, Barberà-Cremades M, Gómez AI, Martín-Sánchez F, Pelegrín P. Macrophage activation and polarization modify P2X7 receptor secretome influencing the inflammatory process. Sci Rep [Internet]. 2016;6. Available from: https://pubmed.ncbi.nlm.nih.gov/26935289/10.1038/srep22586 PMC4776275 26935289

[114] Alarcón-Vila C, Baroja-Mazo A, de Torre-Minguela C, Martínez CM, Martínez-García JJ, Martínez-Banaclocha H, et al. CD14 release induced by P2X7 receptor restricts inflammation and increases survival during sepsis. Elife [Internet]. 2020;9:1–21. 10.7554/eLife.60849 PMC7690950 33135636

[115] Golia MT, Gabrielli M, Verderio C. P2X7 Receptor and extracellular vesicle release. Int J Mol Sci [Internet]. 2023;24. Available from: https://pubmed.ncbi.nlm.nih.gov/37372953/10.3390/ijms24129805 PMC10298639 37372953

[116] Pegoraro A, De Marchi E, Ferracin M, Orioli E, Zanoni M, Bassi C, et al. P2X7 promotes metastatic spreading and triggers release of miRNA-containing exosomes and microvesicles from melanoma cells. Cell Death Dis [Internet]. 2021;12. Available from: https://pubmed.ncbi.nlm.nih.gov/34789738/10.1038/s41419-021-04378-0 PMC8599616 34789738

[117] Pegoraro A, De Marchi E, Ruo L, Zanoni M, Chioccioli S, Caderni G, et al. P2X7 a new therapeutic target to block vesicle-dependent metastasis in colon carcinoma: role of the A2A/CD39/CD73 axis. Cell Death Dis [Internet]. 2025;16. Available from: https://pubmed.ncbi.nlm.nih.gov/40759630/10.1038/s41419-025-07897-2 PMC12322077 40759630

[118] Adinolfi E, De Marchi E, Grignolo M, Szymczak B, Pegoraro A. The P2X7 receptor in oncogenesis and metastatic dissemination: new insights on vesicular release and adenosinergic crosstalk. Int J Mol Sci [Internet]. 2023;24. Available from: https://pubmed.ncbi.nlm.nih.gov/37762206/10.3390/ijms241813906 PMC10531279 37762206

[119] D’Arrigo G, Gabrielli M, Scaroni F, Swuec P, Amin L, Pegoraro A, et al. Astrocytes-derived extracellular vesicles in motion at the neuron surface: Involvement of the prion protein. J Extracell Vesicles [Internet]. 2021;10. Available from: https://pubmed.ncbi.nlm.nih.gov/34276899/10.1002/jev2.12114 PMC8275823 34276899

[120] Welsh JA, Goberdhan DCI, O’Driscoll L, Buzas EI, Blenkiron C, Bussolati B, et al. Minimal information for studies of extracellular vesicles (MISEV2023): From basic to advanced approaches. J Extracell Vesicles [Internet]. 2024;13. Available from: https://pubmed.ncbi.nlm.nih.gov/38326288/10.1002/jev2.12404 PMC10850029 38326288

[121] Falzoni S, Vultaggio-Poma V, Chiozzi P, Tarantini M, Adinolfi E, Boldrini P, et al. The P2X7 receptor is a master regulator of microparticle and mitochondria exchange in mouse microglia. Function [Internet]. 2024;5. Available from: https://pubmed.ncbi.nlm.nih.gov/38984997/10.1093/function/zqae019 PMC11237899 38984997

[122] Vultaggio-Poma V, Falzoni S, Chiozzi P, Sarti AC, Adinolfi E, Giuliani AL, et al. Extracellular ATP is increased by release of ATP-loaded microparticles triggered by nutrient deprivation. Theranostics [Internet]. 2022;12:859–74. 34976217 10.7150/thno.66274 PMC8692914

[123] Thorstenberg ML, Ferreira MVR, Amorim N, Canetti C, Morrone FB, Filho JCA, et al. Purinergic cooperation between P2Y2 and P2X7 receptors promote cutaneous leishmaniasis control: Involvement of pannexin-1 and leukotrienes. Front Immunol [Internet]. 2018;9. Available from: https://pubmed.ncbi.nlm.nih.gov/30038612/10.3389/fimmu.2018.01531 PMC6046465 30038612

[124] Coutinho-Silva R, Perfettini JL, Persechini PM, Dautry-Varsat A, Ojcius DM. Modulation of P2Z/P2X7 receptor activity in macrophages infected with Chlamydia psittaci. Am J Physiol Cell Physiol [Internet]. 2001;280. Available from: https://journals.physiology.org/doi/pdf/10.1152/ajpcell.2001.280.1.C81 10.1152/ajpcell.2001.280.1.C81 11121379

[125] Coutinho-Silva R, Stahl L, Raymond MN, Jungas T, Verbeke P, Burnstock G, et al. Inhibition of chlamydial infectious activity due to P2X 7R-dependent phospholipase D activation. Immun [Internet]. 2003;19:403–12. 10.1016/s1074-7613(03)00235-8 14499115

[126] Chaves MM, Marques-da-Silva C, Monteiro APT, Canetti C, Coutinho-Silva R. Leukotriene B4 Modulates P2X7 Receptor–Mediated Leishmania amazonensis Elimination in Murine Macrophages. J Immunol [Internet]. 2014;192:4765–73. 24729618 10.4049/jimmunol.1301058

[127] Corrêa G, Marques da Silva C, de Abreu Moreira-Souza AC, Vommaro RC, Coutinho-Silva R. Activation of the P2X7 receptor triggers the elimination of Toxoplasma gondii tachyzoites from infected macrophages. Microbes Infect [Internet]. 2010;12:497–504. 20298798 10.1016/j.micinf.2010.03.004

[128] Stoop R, Surprenant A, North RA. Different sensitivities to pH of ATP-induced currents at four cloned P2X receptors. J Neurophysiol [Internet]. 1997;78:1837–40. 9325352 10.1152/jn.1997.78.4.1837

[129] Coddou C, Morales B, González J, Grauso M, Gordillo F, Bull P, et al. Histidine 140 plays a key role in the inhibitory modulation of the P2X 4 nucleotide receptor by copper but not zinc. J Biol Chem [Internet]. 2003;278:36777–85. 12819199 10.1074/jbc.M305177200

[130] Bernier LP, Blais D, Boué-Grabot É, Séguéla P. A dual polybasic motif determines phosphoinositide binding and regulation in the P2X channel family. PLoS One [Internet]. 2012;7. Available from: https://pubmed.ncbi.nlm.nih.gov/22792379/10.1371/journal.pone.0040595 PMC3394732 22792379

[131] Allsopp RC, Lalo U, Evans RJ. Lipid Raft Association and Cholesterol Sensitivity of P2X1-4 Receptors for ATP: chimeras and point mutants identify intracellular amino-terminal residues involved in lipid regulation of P2X1 receptors. J Biol Chem [Internet]. 2010;285:32770. 20699225 10.1074/jbc.M110.148940 PMC2963349

[132] Di Virgilio F, Schmalzing G, Markwardt F. The elusive P2X7 macropore. Trends Cell Biol [Internet]. 2018;28:392–404. 29439897 10.1016/j.tcb.2018.01.005

[133] Yegutkin GG. Adenosine metabolism in the vascular system. Biochem Pharm [Internet]. 2021;187:114373. 33340515 10.1016/j.bcp.2020.114373

[134] Boison D, Yegutkin GG. Adenosine metabolism: emerging concepts for cancer therapy. Cancer Cell [Internet]. 2019;36:582–96. 31821783 10.1016/j.ccell.2019.10.007 PMC7224341

[135] Allard D, Cormery J, Bricha S, Fuselier C, Aghababazadeh FA, Giraud L, et al. Adenosine uptake through the nucleoside transporter ENT1 suppresses antitumor immunity and T-cell pyrimidine synthesis. Cancer Res [Internet]. 2025;85:692–703. 39652568 10.1158/0008-5472.CAN-24-1875

[136] Sanders TJ, Nabel CS, Brouwer M, Hermant AL, Chaible L, Deglasse JP, et al. Inhibition of ENT1 relieves intracellular adenosine-mediated T cell suppression in cancer. Nat Immunol. 2025;26:854–65. 40355731 10.1038/s41590-025-02153-3 PMC12133574

[137] Nims RW, Harbell JW. Best practices for the use and evaluation of animal serum as a component of cell culture medium. Vitr Cell Dev Biol Anim [Internet]. 2017;53:682–90. 10.1007/s11626-017-0184-8 28733930

[138] Young CNJ, Chira N, Róg J, Al-Khalidi R, Benard M, Galas L, et al. Sustained activation of P2X7 induces MMP-2-evoked cleavage and functional purinoceptor inhibition. J Mol Cell Biol [Internet]. 2018;10:229–42. 10.1093/jmcb/mjx030. 28992079 10.1093/jmcb/mjx030

[139] Yegutkin GG, Wieringa B, Robson SC, Jalkanen S. Metabolism of circulating ADP in the bloodstream is mediated via integrated actions of soluble adenylate kinase-1 and NTPDase1/CD39 activities. FASEB J [Internet]. 2012;26:3875–83. 22637533 10.1096/fj.12-205658 PMC3425827

[140] Young CNJ, Górecki DC. P2RX7 purinoceptor as a therapeutic target—the second coming? Front Chem [Internet]. 2018;6. Available from: https://pubmed.ncbi.nlm.nih.gov/30003075/10.3389/fchem.2018.00248 PMC6032550 30003075

[141] Denlinger LC, Angelini G, Schell K, Green DN, Guadarrama AG, Prabhu U, et al. Detection of human P2X7 nucleotide receptor polymorphisms by a novel monocyte pore assay predictive of alterations in lipopolysaccharide-induced cytokine production. J Immunol [Internet]. 2005;174:4424–31. 15778408 10.4049/jimmunol.174.7.4424

[142] Jiang LH, Roger S. Heterologous expression and patch-clamp recording of P2X receptors in HEK293 cells. Methods Mol Biol [Internet]. 2020;2041:261–73. 31646495 10.1007/978-1-4939-9717-6_19

[143] Fischer W, Franke H, Gröger-Arndt H, Illes P. Evidence for the existence of P2Y1,2,4 receptor subtypes in HEK-293 cells: Reactivation of P2Y1 receptors after repetitive agonist application. Naunyn Schmiedebergs Arch Pharm [Internet]. 2005;371:466–72. 10.1007/s00210-005-1070-6 16025270

[144] Benzaquen J, Dit Hreich SJ, Heeke S, Juhel T, Lalvee S, Bauwens S, et al. P2RX7B is a new theranostic marker for lung adenocarcinoma patients. Theranostics [Internet]. 2020;10:10849–60. 33042257 10.7150/thno.48229 PMC7532666

[145] Cheewatrakoolpong B, Gilchrest H, Anthes JC, Greenfeder S. Identification and characterization of splice variants of the human P2X7 ATP channel. Biochem Biophys Res Commun [Internet]. 2005;332:17–27. 15896293 10.1016/j.bbrc.2005.04.087

[146] Gilbert S, Oliphant C, Hassan S, Peille A, Bronsert P, Falzoni S, et al. ATP in the tumour microenvironment drives expression of nfP2X7, a key mediator of cancer cell survival. Oncogene. 2018;38:194–208. 30087439 10.1038/s41388-018-0426-6 PMC6328436

[147] Giuliani AL, Colognesi D, Ricco T, Roncato C, Capece M, Amoroso F, et al. Trophic activity of human P2X7 receptor isoforms A and B in osteosarcoma. PLoS One [Internet]. 2014;9. Available from: https://pubmed.ncbi.nlm.nih.gov/25226385/10.1371/journal.pone.0107224 PMC4165768 25226385

[148] Ulrich H, Ratajczak MZ, Schneider G, Adinolfi E, Orioli E, Ferrazoli EG, et al. Kinin and purine signaling contributes to neuroblastoma metastasis. Front Pharmacol [Internet]. 2018;9. Available from: https://pubmed.ncbi.nlm.nih.gov/29867502/10.3389/fphar.2018.00500 PMC5968427 29867502

[149] Arnaud-Sampaio VF, Bento CA, Glaser T, Adinolfi E, Ulrich H, Lameu C. P2X7 receptor isoform B is a key drug resistance mediator for neuroblastoma. Front Oncol [Internet]. 2022;12. Available from: https://pubmed.ncbi.nlm.nih.gov/36091161/10.3389/fonc.2022.966404 PMC9458077 36091161

[150] Gilbert SM, Gidley Baird A, Glazer S, Barden JA, Glazer A, Teh LC, et al. A phase I clinical trial demonstrates that nfP2X7-targeted antibodies provide a novel, safe and tolerable topical therapy for basal cell carcinoma. Br J Dermatol [Internet]. 2017;177:117–24. 10.1111/bjd.15364. 28150889 10.1111/bjd.15364

[151] Bandara V, Foeng J, Gundsambuu B, Norton TS, Napoli S, McPeake DJ, et al. Pre-clinical validation of a pan-cancer CAR-T cell immunotherapy targeting nfP2X7. Nat Commun [Internet]. 2023;14:5546. 37684239 10.1038/s41467-023-41338-y PMC10491676

[152] Arbeloa J, Pérez-Samartín A, Gottlieb M, Matute C. P2X7 receptor blockade prevents ATP excitotoxicity in neurons and reduces brain damage after ischemia. Neurobiol Dis [Internet]. 2012;45:954–61. 22186422 10.1016/j.nbd.2011.12.014

[153] Matute C, Torre I, Pérez-Cerdá F, Pérez-Samartín A, Alberdi E, Etxebarria E, et al. P2X7 Receptor blockade prevents ATP excitotoxicity in oligodendrocytes and ameliorates experimental autoimmune encephalomyelitis. J Neurosci [Internet]. 2007;27:9525–33. Available from: https://www.jneurosci.org/content/27/35/9525 10.1523/JNEUROSCI.0579-07.2007 PMC6673129 17728465

[154] Cisneros-Mejorado A, Pérez-Samartín A, Gottlieb M, Matute C. ATP Signaling in brain: release, excitotoxicity and potential therapeutic targets. Cell Mol Neurobiol [Internet]. 2015;35:1–6. Available from: https://link.springer.com/article/10.1007/s10571-014-0092-3 10.1007/s10571-014-0092-3 PMC11488175 25096398

[155] Domercq M, Perez-Samartin A, Aparicio D, Alberdi E, Pampliega O, Matute C. P2X7 receptors mediate ischemic damage to oligodendrocytes. Glia [Internet]. 2010;58:730–40. 20029962 10.1002/glia.20958

[156] Montilla A, Zabala A, Matute C, Domercq M. Functional and metabolic characterization of microglia culture in a defined medium. Front Cell Neurosci [Internet]. 2020;14. Available from: https://pubmed.ncbi.nlm.nih.gov/32116565/10.3389/fncel.2020.00022 PMC7025516 32116565

[157] Ribeiro DE, Glaser T, Oliveira-Giacomelli, Ulrich H. Purinergic receptors in neurogenic processes. Brain Res Bull [Internet]. 2019;151:3–11. https://pubmed.ncbi.nlm.nih.gov/30593881/Sep 1 [cited 2025 Apr 7]Available from.. 10.1016/j.brainresbull.2018.12.013 30593881

[158] Negraes PD, Schwindt TT, Trujillo CA, Ulrich H. Neural differentiation of P19 carcinoma cells and primary neurospheres: cell morphology, proliferation, viability, and functionality. Curr Protoc Stem Cell Biol [Internet]. 2012;(Suppl.20). Available from: https://pubmed.ncbi.nlm.nih.gov/22415841/10.1002/9780470151808.sc02d09s20 22415841

[159] Brass D, Grably MR, Bronstein-Sitton N, Gohar O, Meir A. Using antibodies against P2Y and P2X receptors in purinergic signaling research. Purinergic Signal [Internet]. 2011;8:61. 22086554 10.1007/s11302-011-9278-z PMC3265709

[160] Arnaud-Sampaio VF, Rabelo ILA, Bento CA, Glaser T, Bezerra J, Coutinho-Silva R. et al. Using cytometry for investigation of purinergic signaling in tumor-associated macrophages. Cytom A [Internet. 2020;97:1109–26. 10.1002/cyto.a.24035 32633884

[161] Koziol-White C, Gebski E, Cao G, Panettieri RA. Precision cut lung slices: an integrated ex vivo model for studying lung physiology, pharmacology, disease pathogenesis and drug discovery. Respir Res [Internet]. 2024;25:1–21. 38824592 10.1186/s12931-024-02855-6 PMC11144351

[162] Wu X, van Dijk EM, Bos IST, Kistemaker LEM, Gosens R. Mouse lung tissue slice culture. Methods Mol Biol [Internet]. 2019;1940:297–311. 30788834 10.1007/978-1-4939-9086-3_21

[163] De Marchi E, Pegoraro A, Turiello R, Di Virgilio F, Morello S, Adinolfi E. A2A Receptor contributes to tumor progression in P2X7 null mice. Front Cell Dev Biol [Internet]. 2022;10. Available from: https://pubmed.ncbi.nlm.nih.gov/35663396/10.3389/fcell.2022.876510 PMC9159855 35663396

[164] Yan J, Li XY, Aguilera AR, Xiao C, Jacoberger-Foissac C, Nowlan B, et al. Control of Metastases via Myeloid CD39 and NK Cell Effector Function. Cancer Immunol Res [Internet]. 2020;8:356–67. 31992567 10.1158/2326-6066.CIR-19-0749

[165] Gölöncsér F, Baranyi M, Tod P, Maácz F, Sperlágh B. P2X7 receptor inhibition alleviates mania-like behavior independently of interleukin-1β. iScience [Internet]. 2024;27. Available from: https://pubmed.ncbi.nlm.nih.gov/38444608/10.1016/j.isci.2024.109284 PMC10914489 38444608

[166] Bhattacharya A, Wang Q, Ao H, Shoblock JR, Lord B, Aluisio L, et al. Pharmacological characterization of a novel centrally permeable P2X7 receptor antagonist: JNJ-47965567. Br J Pharm [Internet]. 2013;170:624–40. 10.1111/bph.12314 PMC3792000 23889535

[167] Csölle C, Baranyi M, Zsilla G, Kittel Á, Gölöncsér F, Illes P, et al. Neurochemical changes in the mouse hippocampus underlying the antidepressant effect of genetic deletion of P2X7 receptors. PLoS One [Internet]. 2013;8. Available from: https://pubmed.ncbi.nlm.nih.gov/23805233/10.1371/journal.pone.0066547 PMC3689833 23805233

[168] Jarvis MF, Burgard EC, McGaraughty S, Honore P, Lynch K, Brennan TJ, et al. A-317491, a novel potent and selective non-nucleotide antagonist of P2X3 and P2X2/3 receptors, reduces chronic inflammatory and neuropathic pain in the rat. Proc Natl Acad Sci USA [Internet]. 2002;99:17179. 12482951 10.1073/pnas.252537299 PMC139289

[169] Wu G, Whiteside GT, Lee G, Nolan S, Niosi M, Pearson MS, et al. A-317491, a selective P2X 3/P2X 2/3 receptor antagonist, reverses inflammatory mechanical hyperalgesia through action at peripheral receptors in rats. Eur J Pharm [Internet]. 2004;504:45–53. 10.1016/j.ejphar.2004.09.056 15507220

[170] Gilabert D, Duveau A, Carracedo S, Linck N, Langla A, Muramatsu R, et al. Microglial P2X4 receptors are essential for spinal neurons hyperexcitability and tactile allodynia in male and female neuropathic mice. iScience [Internet]. 2023;26. Available from: https://pubmed.ncbi.nlm.nih.gov/37860691/10.1016/j.isci.2023.108110 PMC10583052 37860691

[171] Long T, He W, Pan Q, Zhang S, Zhang Y, Liu C, et al. Microglia P2X4 receptor contributes to central sensitization following recurrent nitroglycerin stimulation. J Neuroinflamm [Internet]. 2018;15. Available from: https://pubmed.ncbi.nlm.nih.gov/30165876/10.1186/s12974-018-1285-3 PMC6117935 30165876

[172] Amoroso F, Capece M, Rotondo A, Cangelosi D, Ferracin M, Franceschini A, et al. The P2X7 receptor is a key modulator of the PI3K/GSK3β/VEGF signaling network: evidence in experimental neuroblastoma. Oncogene [Internet]. 2015;34:5240–51. 25619831 10.1038/onc.2014.444

[173] Adinolfi E, Raffaghello L, Giuliani AL, Cavazzini L, Capece M, Chiozzi P, et al. Expression of P2X7 receptor increases in vivo tumor growth. Cancer Res [Internet]. 2012;72:2957–69. 22505653 10.1158/0008-5472.CAN-11-1947

[174] Fortuny-Gomez A, Fountain SJ. Pharmacological differences between human and mouse P2X4 receptor explored using old and new tools. Purinergic Signal [Internet]. 2024;20:659–67. 38767821 10.1007/s11302-024-10018-x PMC11554605

[175] Al-Khalidi R, Panicucci C, Cox P, Chira N, Róg J, Young CNJ, et al. Zidovudine ameliorates pathology in the mouse model of Duchenne muscular dystrophy via P2RX7 purinoceptor antagonism. Acta Neuropathol Commun [Internet]. 2018;6:27 https://pubmed.ncbi.nlm.nih.gov/29642926/. 29642926 10.1186/s40478-018-0530-4 PMC5896059

[176] Rumney RMH, Górecki DC. Knockout and knock-in mouse models to study purinergic signaling. In: Methods in molecular biology [Internet]. 2020:17–43. Available from: http://link.springer.com/10.1007/978-1-4939-9717-6_2 10.1007/978-1-4939-9717-6_2 31646478

[177] Chessell IP, Hatcher JP, Bountra C, Michel AD, Hughes JP, Green P, et al. Disruption of the P2X7 purinoceptor gene abolishes chronic inflammatory and neuropathic pain. Pain [Internet]. 2005;114:386–96. 15777864 10.1016/j.pain.2005.01.002

[178] Basso AM, Bratcher NA, Harris RR, Jarvis MF, Decker MW, Rueter LE. Behavioral profile of P2X7 receptor knockout mice in animal models of depression and anxiety: Relevance for neuropsychiatric disorders. Behav Brain Res [Internet]. 2009;198:83–90. Available from: https://www.sciencedirect.com/science/article/abs/pii/S0166432808005718 10.1016/j.bbr.2008.10.018 18996151

[179] Apolloni S, Amadio S, Parisi C, Matteucci A, Potenza RL, Armida M, et al. Spinal cord pathology is ameliorated by P2X7 antagonism in a SOD1-mutant mouse model of amyotrophic lateral sclerosis. DMM Dis Models Mech [Internet]. 2014;7:1101–9. 10.1242/dmm.017038 PMC4142730 25038061

[180] Novak I, Jans IM, Wohlfahrt L. Effect of P2X7 receptor knockout on exocrine secretion of pancreas, salivary glands and lacrimal glands. J Physiol [Internet]. 2010;588:3615–27. 20643770 10.1113/jphysiol.2010.190017 PMC2988522

[181] Adriouch S, Dox C, Welge V, Seman M, Koch-Nolte F, Haag F. Cutting edge: a natural P451L mutation in the cytoplasmic domain impairs the function of the mouse P2X7 receptor. J Immunol [Internet]. 2002;169:4108–12. 12370338 10.4049/jimmunol.169.8.4108

[182] Engel T, Gomez-Villafuertes R, Tanaka K, Mesuret G, Sanz-Rodriguez A, Garcia-Huerta P, et al. Seizure suppression and neuroprotection by targeting the purinergic P2X7 receptor during status epilepticus in mice. FASEB J [Internet]. 2012;26:1616–28. 22198387 10.1096/fj.11-196089

[183] Kaczmarek-Hajek K, Zhang J, Kopp R, Grosche A, Rissiek B, Saul A, et al. Re-evaluation of neuronal P2X7 expression using novel mouse models and a P2X7-specific nanobody. Elife [Internet]. 2018;7. Available from: https://pubmed.ncbi.nlm.nih.gov/30074479/10.7554/eLife.36217 PMC6140716 30074479

[184] Ramírez-Fernández A, Urbina-Treviño L, Conte G, Alves M, Rissiek B, Durner A, et al. Deviant reporter expression and P2X4 passenger gene overexpression in the soluble EGFP BAC transgenic P2X7 reporter mouse model. Sci Rep [Internet]. 2020;10:19876. 33199725 10.1038/s41598-020-76428-0 PMC7669894

[185] Cockayne DA, Hamilton SG, Zhu QM, Dunn PM, Zhong Y, Novakovic S, et al. Urinary bladder hyporeflexia and reduced pain-related behaviour in P2X3-deficient mice. Nat [Internet]. 2000;407:1011–5. 10.1038/35039519 11069181

[186] Souslova V, Cesare P, Ding Y, Akopian AN, Stanfa L, Suzuki R, et al. Warm-coding deficits and aberrant inflammatory pain in mice lacking P2X3 receptors. Nat [Internet]. 2000;407:1015–7. 10.1038/35039526 11069182

[187] Deiteren A, Van Der Linden L, De Wit A, Ceuleers H, Buckinx R, Timmermans JP, et al. P2X3 receptors mediate visceral hypersensitivity during acute chemically-induced colitis and in the post-inflammatory phase via different mechanisms of sensitization. PLoS One [Internet]. 2015;10. Available from: https://pubmed.ncbi.nlm.nih.gov/25885345/10.1371/journal.pone.0123810 PMC4401691 25885345

[188] Tsuda M, Kuboyama K, Inoue T, Nagata K, Tozaki-Saitoh H, Inoue K. Behavioral phenotypes of mice lacking purinergic P2X4 receptors in acute and chronic pain assays. Mol Pain [Internet]. 2009;5:28. 19515262 10.1186/1744-8069-5-28 PMC2704200

[189] Bou Sader Nehme S, Sanchez-Sarasua S, Adel R, Tuifua M, Ali A, Essawy AE, et al. P2X4 signalling contributes to hyperactivity but not pain sensitization comorbidity in a mouse model of attention deficit/hyperactivity disorder. Front Pharmacol [Internet]. 2024;14. Available from: https://pubmed.ncbi.nlm.nih.gov/38239187/10.3389/fphar.2023.1288994 PMC10794506 38239187

[190] Ferrari D, Chiozzi P, Falzoni S, Dal Susino M, Collo G, Buell G, et al. ATP-mediated cytotoxicity in microglial cells. Neuropharmacol [Internet]. 1997;36:1295–301. 10.1016/s0028-3908(97)00137-8 9364484

[191] Di Virgilio F, Dal Ben D, Sarti AC, Giuliani AL, Falzoni S. The P2X7 receptor in infection and inflammation. Immun [Internet]. 2017;47:15–31. 10.1016/j.immuni.2017.06.020 28723547

[192] Orioli E, De Marchi E, Giuliani AL, Adinolfi E. P2X7 Receptor orchestrates multiple signalling pathways triggering inflammation, autophagy and metabolic/trophic responses. Curr Med Chem [Internet]. 2017;24. Available from: https://pubmed.ncbi.nlm.nih.gov/28266268/10.2174/0929867324666170303161659 28266268

[193] Tsao HK, Chiu PH, Sun SH. PKC-dependent ERK phosphorylation is essential for P2X7 receptor-mediated neuronal differentiation of neural progenitor cells. Cell Death Dis [Internet]. 2013;4. Available from: https://pubmed.ncbi.nlm.nih.gov/23907465/10.1038/cddis.2013.274 PMC3763436 23907465

[194] Rigato C, Swinnen N, Buckinx R, Couillin I, Mangin JM, Rigo JM, et al. Microglia proliferation is controlled by P2X7 receptors in a pannexin-1-independent manner during early embryonic spinal cord invasion. J Neurosci [Internet]. 2012;32:11559. 22915101 10.1523/JNEUROSCI.1042-12.2012 PMC6703767

[195] Illes P, Khan TM, Rubini P. Neuronal P2X7 receptors revisited: do they really exist?. J Neurosci [Internet]. 2017;37:7049–62. 28747388 10.1523/JNEUROSCI.3103-16.2017 PMC6705732

[196] Teresa Miras-Portugal PM, Sebastián-Serrano Á, De Diego García L, Díaz-Hernández M. Neuronal P2X7 receptor: Involvement in neuronal physiology and. J Neurosci [Internet]. 2017;37:7063–72. 28747389 10.1523/JNEUROSCI.3104-16.2017 PMC6705729

[197] Alves M, Gil B, Villegas-Salmerón J, Salari V, Martins-Ferreira R, Arribas Blázquez M, et al. Opposing effects of the purinergic P2X7 receptor on seizures in neurons and microglia in male mice. Brain Behav Immun [Internet]. 2024;120:121–40. 38777288 10.1016/j.bbi.2024.05.023

[198] Pegoraro A, Grignolo M, Ruo L, Ricci L, Adinolfi E. P2X7 Variants in Pathophysiology. Int J Mol Sci. 2024; 25:6673 [Internet]. 2024;25(12):6673. Available from: https://www.mdpi.com/1422-0067/25/12/6673/htm 10.3390/ijms25126673 PMC11204217 38928378

[199] Soni S, Lukhey MS, Thawkar BS, Chintamaneni M, Kaur G, Joshi H, et al. A current review on P2X7 receptor antagonist patents in the treatment of neuroinflammatory disorders: a patent review on antagonists. Naunyn Schmiedebergs Arch Pharm [Internet]. 2024;397:4643–56. 10.1007/s00210-024-02994-z 38349395

[200] Iqbal J, Bano S, Khan IA, Huang Q. A patent review of P2X7 receptor antagonists to treat inflammatory diseases (2018–present). Expert Opin Ther Pat [Internet]. 2024;34:263–71. 38828613 10.1080/13543776.2024.2363885

[201] Adinolfi E, De Marchi E, Orioli E, Pegoraro A, Di Virgilio F. Role of the P2X7 receptor in tumor-associated inflammation. Curr Opin Pharm [Internet]. 2019;47:59–64. 10.1016/j.coph.2019.02.012 30921559

[202] Andrejew R, Oliveira-Giacomelli Á, Ribeiro DE, Glaser T, Arnaud-Sampaio VF, Lameu C, et al. The P2X7 receptor: central hub of brain diseases. Front Mol Neurosci [Internet]. 2020;13. Available from: https://pubmed.ncbi.nlm.nih.gov/32848594/10.3389/fnmol.2020.00124 PMC7413029 32848594

[203] Zhou J, Zhou Z, Liu X, Yin HY, Tang Y, Cao X. P2X7 receptor–mediated inflammation in cardiovascular disease. Front Pharm [Internet]. 2021;12:654425. 10.3389/fphar.2021.654425 PMC8117356 33995071

[204] Profaci CP, Munji RN, Pulido RS, Daneman R. The blood–brain barrier in health and disease: Important unanswered questions. Journal of Experimental Medicine [Internet]. 2020;217. Available from: https://pubmed.ncbi.nlm.nih.gov/32211826/10.1084/jem.20190062 PMC7144528 32211826

[205] Illes P, Rubini P, Huang L, Tang Y. The P2X7 receptor: a new therapeutic target in Alzheimer’s disease. Expert Opin Ther Targets [Internet]. 2019;23:165–76. https://pubmed.ncbi.nlm.nih.gov/30691318/Mar 4 [cited 2025 Sep 8]Available from. 30691318 10.1080/14728222.2019.1575811

[206] Beamer E, Kuchukulla M, Boison D, Engel T. ATP and adenosine—two players in the control of seizures and epilepsy development. Prog Neurobiol [Internet]. 2021;204. Available from: https://pubmed.ncbi.nlm.nih.gov/34144123/10.1016/j.pneurobio.2021.102105 PMC10237002 34144123

[207] Metzger MW, Walser SM, Aprile-Garcia F, Dedic N, Chen A, Holsboer F, et al. Genetically dissecting P2rx7 expression within the central nervous system using conditional humanized mice. Purinergic Signal [Internet]. 2017;13:153–70. 27858314 10.1007/s11302-016-9546-z PMC5432476

[208] Stamataki M, Rissiek B, Magnus T, Körbelin J. Microglia targeting by adeno-associated viral vectors. Front Immunol. 2024;15:1425892. Jul 5. 39035004 10.3389/fimmu.2024.1425892 PMC11257843

[209] Toader C, Dumitru AV, Eva L, Serban M, Covache-Busuioc RA, Ciurea AV. Nanoparticle strategies for treating CNS disorders: a comprehensive review of drug delivery and theranostic applications. Int J Mol Sci. 2024;25:13302. 39769066 10.3390/ijms252413302 PMC11676454

[210] Zhao XF, Alam MM, Liao Y, Huang T, Mathur R, Zhu X. et al. Targeting microglia using Cx3cr1-Cre lines: revisiting the specificity. eNeuro [Internet. 2019;6:1–11. 10.1523/ENEURO.0114-19.2019 PMC6620394 31201215

[211] Illes P, Xu GY, Tang Y. Purinergic signaling in the central nervous system in health and disease. Neurosci Bull [Internet]. 2020;36:1239–41. 33146814 10.1007/s12264-020-00602-7 PMC7674523

[212] Ren W, Rubini P, Tang Y, Engel T, Illes P. Inherent p2x7 receptors regulate macrophage functions during inflammatory diseases. Int J Mol Sci [Internet]. 2022;23. Available from: https://pubmed.ncbi.nlm.nih.gov/35008658/10.3390/ijms23010232 PMC8745241 35008658

[213] Bradley JE, Ramirez G, Hagood JS. Roles and regulation of Thy-1, a context-dependent modulator of cell phenotype. BioFactors [Internet]. 2009;35:258–65. 19422052 10.1002/biof.41 PMC5675016

[214] Li X, Du ZJ, Chen MQ, Chen JJ, Liang ZM, Ding XT, et al. The effects of tamoxifen on mouse behavior. Genes Brain Behav [Internet]. 2020;19. Available from: https://pubmed.ncbi.nlm.nih.gov/31652391/10.1111/gbb.12620 31652391

[215] Turner PV, Pang DSJ, Lofgren JLS. A review of pain assessment methods in laboratory rodents. Comp Med [Internet]. 2019;69:451–67. 31896391 10.30802/AALAS-CM-19-000042 PMC6935698

[216] Otrokocsi L, Kittel Á, Sperlágh B. P2X7 receptors drive spine synapse plasticity in the learned helplessness model of depression. Int J Neuropsychopharmacol [Internet]. 2017;20:813–22. 28633291 10.1093/ijnp/pyx046 PMC5632310

[217] Petković A, Chaudhury D. Encore: behavioural animal models of stress, depression and mood disorders. Front Behav Neurosci [Internet]. 2022;16. Available from: https://pubmed.ncbi.nlm.nih.gov/36004305/10.3389/fnbeh.2022.931964 PMC9395206 36004305

[218] Ghafarimoghadam M, Mashayekh R, Gholami M, Fereydani P, Shelley-Tremblay J, Kandezi N, et al. A review of behavioral methods for the evaluation of cognitive performance in animal models: Current techniques and links to human cognition. Physiol Behav [Internet]. 2022;244:113652. 34801559 10.1016/j.physbeh.2021.113652

[219] Jiang LH, Mackenzie AB, North RA, Surprenant A. Brilliant blue G selectively blocks ATP-gated rat P2X7 receptors. Mol Pharm. 2000;58:82–8. 10860929

[220] Bhattacharya A. Recent advances in CNS P2X7 physiology and pharmacology: focus on neuropsychiatric disorders. Front Pharmacol [Internet]. 2018;9. Available from: https://pubmed.ncbi.nlm.nih.gov/29449810/10.3389/fphar.2018.00030 PMC5799703 29449810

[221] du Sert NP, Hurst V, Ahluwalia A, Alam S, Avey MT, Baker M, et al. The ARRIVE guidelines 2.0: Updated guidelines for reporting animal research. PLoS Biol [Internet]. 2020;18. Available from: https://pubmed.ncbi.nlm.nih.gov/32663219/10.1371/journal.pbio.3000410 PMC7360023 32663219

[222] Festing MFW, Altman DG. Guidelines for the design and statistical analysis of experiments using laboratory animals. ILAR J [Internet]. 2002;43:244–57. 12391400 10.1093/ilar.43.4.244

[223] Csölle C, Andó RD, Kittel Á, Gölöncsér F, Baranyi M, Soproni K, et al. The absence of P2X7 receptors (P2rx7) on non-haematopoietic cells leads to selective alteration in mood-related behaviour with dysregulated gene expression and stress reactivity in mice. Int J Neuropsychopharmacol [Internet]. 2013;16:213–33. 22243662 10.1017/S1461145711001933 PMC3666310

[224] Ribeiro DE, Roncalho AL, Glaser T, Ulrich H, Wegener G, Joca S. P2X7 receptor signaling in stress and depression. Int J Mol Sci [Internet]. 2019;20. Available from: https://pubmed.ncbi.nlm.nih.gov/31174279/10.3390/ijms20112778 PMC6600521 31174279

[225] Cisneros-Mejorado AJ, Pérez-Samartín A, Domercq M, Arellano RO, Gottlieb M, Koch-Nolte F, et al. P2X7 receptors as a therapeutic target in cerebrovascular diseases. Front Mol Neurosci [Internet]. 2020;13. Available from: https://pubmed.ncbi.nlm.nih.gov/32714144/10.3389/fnmol.2020.00092 PMC7340211 32714144

[226] Rawish E, Langer HF. Platelets and the role of P2X receptors in nociception, pain, neuronal toxicity and thromboinflammation. Int J Mol Sci. 2022;23:6585 . 35743029 10.3390/ijms23126585 PMC9224425

[227] Challa SR, Levingston H, Fornal CA, Baker IM, Boston J, Shanthappa N, et al. Temporal mRNA expression of purinergic P2 receptors in the brain following cerebral ischemia and reperfusion: similarities and distinct variations between rats and mice. Int J Mol Sci [Internet]. 2025;26. Available from: https://pubmed.ncbi.nlm.nih.gov/40141023/10.3390/ijms26062379 PMC11941906 40141023

[228] Bernal-Chico A, Manterola A, Cipriani R, Katona I, Matute C, Mato S. P2x7 receptors control demyelination and inflammation in the cuprizone model. Brain Behav Immun Health [Internet]. 2020;4. Available from: https://pubmed.ncbi.nlm.nih.gov/34589847/10.1016/j.bbih.2020.100062 PMC8474271 34589847

[229] Zabala A, Vazquez-Villoldo N, Rissiek B, Gejo J, Martin A, Palomino A, et al. P2X4 receptor controls microglia activation and favors remyelination in autoimmune encephalitis. EMBO Mol Med [Internet]. 2018;10:8743 . 10.15252/emmm.201708743 PMC6079537 29973381

[230] Montilla A, Zabala A, Calvo I, Bosch-Juan M, Tomé-Velasco I, Mata P, et al. Microglia regulate myelin clearance and cholesterol metabolism after demyelination via interferon regulatory factor 5. Cell Mol Life Sci [Internet]. 2025;82:1–21. 10.1007/s00018-025-05648-2 PMC11947375 40137979

[231] Zhou W, Xia S, Wang C, Yang Q, Verkhratsky A, Niu J. Critical analysis of translational potential of rodent models of white matter pathology across a wide spectrum of human diseases. Cell Death Dis. 2025;16:1 [Internet]. 2025;16:1–26. https://www.nature.com/articles/s41419-025-07893-6 10.1038/s41419-025-07893-6 PMC12313980 40744926

[232] Janho dit Hreich S, Juhel T, Hofman P, Vouret-Craviari V. Protocol for evaluating in vivo the activation of the P2RX7 immunomodulator. Biol Proced Online [Internet]. 2023;25. Available from: https://pubmed.ncbi.nlm.nih.gov/36600200/10.1186/s12575-022-00188-6 PMC9811721 36600200

[233] Schwarz N, Fliegert R, Adriouch S, Seman M, Guse AH, Haag F, et al. Activation of the P2X7 ion channel by soluble and covalently bound ligands. Purinergic Signal [Internet]. 2009;5:139 . 19255877 10.1007/s11302-009-9135-5 PMC2686825

[234] Hong S, Schwarz N, Brass A, Seman M, Haag F, Koch-Nolte F, et al. Differential regulation of P2X7 receptor activation by extracellular nicotinamide adenine dinucleotide and ecto-ADP-ribosyltransferases in murine macrophages and T cells. J Immunol [Internet]. 2009;183:578–92. 19542469 10.4049/jimmunol.0900120 PMC2768492

[235] Stähler T, Danquah W, Demeules M, Gondé H, Hardet R, Haag F, et al. Development of antibody and nanobody tools for P2X7. Methods Mol Biol [Internet]. 2022;2510:99–127. 35776322 10.1007/978-1-0716-2384-8_6

[236] Danquah W, Catherine MS, Rissiek B, Pinto C, Arnau SP, Amadi M, et al. Nanobodies that block gating of the P2X7 ion channel ameliorate inflammation. Sci Transl Med [Internet]. 2016;8. Available from: https://pubmed.ncbi.nlm.nih.gov/27881823/10.1126/scitranslmed.aaf8463 27881823

[237] Rissiek B, Haag F, Boyer O, Koch-Nolte F, Adriouch S. ADP-ribosylation of P2X7: a matter of life and death for regulatory T cells and natural killer T cells. Curr Top Microbiol Immunol [Internet]. 2015;384:107–26. 25048544 10.1007/82_2014_420

[238] Adriouch S, Bannas P, Schwarz N, Fliegert R, Guse AH, Seman M, et al. ADP-ribosylation at R125 gates the P2X7 ion channel by presenting a covalent ligand to its nucleotide binding site. FASEB J [Internet]. 2008;22:861–9. 17928361 10.1096/fj.07-9294com

[239] Hubert S, Rissiek B, Klages K, Huehn J, Sparwasser T, Haag F, et al. Extracellular NAD+ shapes the Foxp3+ regulatory T cell compartment through the ART2-P2X7 pathway. J Exp Med [Internet]. 2010;207:2561–8. 20975043 10.1084/jem.20091154 PMC2989765

[240] Seman M, Adriouch S, Scheuplein F, Krebs C, Freese D, Glowacki G, et al. NAD-induced T cell death: ADP-ribosylation of cell surface proteins by ART2 activates the cytolytic P2X7 purinoceptor. Immun [Internet]. 2003;19:571–82. 10.1016/s1074-7613(03)00266-8 14563321

[241] Bannas P, Adriouch S, Kahl S, Braasch F, Haag F, Koch-Nolte F. Activity and specificity of toxin-related mouse T cell ecto-ADP-ribosyltransferase ART2.2 depends on its association with lipid rafts. Blood [Internet]. 2005;105:3663–70. 15657180 10.1182/blood-2004-08-3325

[242] Haag F, Adriouch S, Braß A, Jung C, Möller S, Scheuplein F, et al. Extracellular NAD and ATP: Partners in immune cell modulation. Purinergic Signal [Internet]. 2007;3:71–81. 18404420 10.1007/s11302-006-9038-7 PMC2096762

[243] Sainz RM, Rodriguez-Quintero JH, Maldifassi MC, Stiles BM, Wennerberg E. Tumour immune escape via P2X7 receptor signalling. Front Immunol. 2023;14:1287310. Oct 30. 38022596 10.3389/fimmu.2023.1287310 PMC10643160

[244] Wennerberg E, Mukherjee S, Sainz RM, Stiles BM. The ART of tumor immune escape. Oncoimmunology [Internet]. 2022;11. Available from: https://pubmed.ncbi.nlm.nih.gov/35602287/10.1080/2162402X.2022.2076310 PMC9116389 35602287

[245] Wennerberg E, Mukherjee S, Spada S, Hung C, Agrusa CJ, Chen C, et al. Expression of the mono-ADP-ribosyltransferase ART1 by tumor cells mediates immune resistance in non-small cell lung cancer. Sci Transl Med [Internet]. 2022;14. Available from: https://pubmed.ncbi.nlm.nih.gov/35294260/10.1126/scitranslmed.abe8195 PMC9256502 35294260

[246] Adriouch S, Hubert S, Pechberty S, Koch-Nolte F, Haag F, Seman M. NAD+ released during inflammation participates in T cell homeostasis by inducing ART2-mediated death of naive T cells in vivo. J Immunol [Internet]. 2007;179:186–94. 17579037 10.4049/jimmunol.179.1.186

[247] Schwarz N, Drouot L, Nicke A, Fliegert R, Boyer O, Guse AH, et al. Alternative splicing of the N-terminal cytosolic and transmembrane domains of P2X7 controls gating of the ion channel by ADP-ribosylation. PLoS One [Internet]. 2012;7. https://pubmed.ncbi.nlm.nih.gov/22848454/10.1371/journal.pone.0041269 PMC3407210 22848454

[248] Menzel S, Rissiek B, Bannas P, Jakoby T, Miksiewicz M, Schwarz N, et al. Nucleotide-induced membrane-proximal proteolysis controls the substrate specificity of T cell Ecto-ADP-ribosyltransferase ARTC2.2. J Immunol [Internet]. 2015;195:2057–66. 26209623 10.4049/jimmunol.1401677

[249] Junge M, Liaukouskaya N, Schwarz N, Pinto-Espinoza C, Schaffrath AZ, Rissiek B, et al. ATP-gated P2X7-Ion channel on kidney-resident natural killer T cells and memory T cells in intrarenal inflammation. J Am Soc Nephrol [Internet]. 2025;36:602–13. 39675762 10.1681/ASN.0000000564 PMC11975244

[250] Rissiek B, Danquah W, Haag F, Koch-Nolte F. Technical Advance: a new cell preparation strategy that greatly improves the yield of vital and functional Tregs and NKT cells. J Leukoc Biol [Internet]. 2014;95:543–9. 24212099 10.1189/jlb.0713407

[251] Koch-Nolte F, Reyelt J, Schößow B, Schwarz N, Scheuplein F, Rothenburg S, et al. Single domain antibodies from llama effectively and specifically block T cell ecto-ADP-ribosyltransferase ART2.2 in vivo. FASEB J [Internet]. 2007;21:3490–8. 17575259 10.1096/fj.07-8661com

[252] Gondé H, Demeules M, Hardet R, Scarpitta A, Junge M, Pinto-Espinoza C, et al. A methodological approach using rAAV vectors encoding nanobody-based biologics to evaluate ARTC2.2 and P2X7 in vivo. Front Immunol [Internet]. 2021;12. Available from: https://pubmed.ncbi.nlm.nih.gov/34489954/10.3389/fimmu.2021.704408 PMC8417108 34489954

[253] Möller S, Jung C, Adriouch S, Dubberke G, Seyfried F, Seman M, et al. Monitoring the expression of purinoceptors and nucleotide-metabolizing ecto-enzymes with antibodies directed against proteins in native conformation. Purinergic Signal [Internet]. 2007;3:359 . 18404449 10.1007/s11302-007-9084-9 PMC2072918

[254] Koch-Nolte F, Eichhoff A, Pinto-Espinoza C, Schwarz N, Schäfer T, Menzel S, et al. Novel biologics targeting the P2X7 ion channel. Curr Opin Pharm [Internet]. 2019;47:110–8. 10.1016/j.coph.2019.03.001 30986625

[255] Menzel S, Schwarz N, Haag F, Koch-Nolte F. Nanobody-based biologics for modulating purinergic signaling in inflammation and immunity. Front Pharmacol. 2018;9. 10.3389/fphar.2018.00266 PMC5880931 29636685

[256] Adriouch S, Dubberke G, Diessenbacher P, Rassendren F, Seman M, Haag F, et al. Probing the expression and function of the P2X7 purinoceptor with antibodies raised by genetic immunization. Cell Immunol [Internet]. 2005;236:72–7. 16165114 10.1016/j.cellimm.2005.08.011

[257] Eichhoff AM, Börner K, Albrecht B, Schäfer W, Baum N, Haag F, et al. Nanobody-enhanced targeting of AAV gene therapy vectors. Mol Ther Methods Clin Dev [Internet]. 2019;15:211–20. 31687421 10.1016/j.omtm.2019.09.003 PMC6819893

[258] Eggers M, Rühl F, Haag F, Koch-Nolte F. Nanobodies as probes to investigate purinergic signaling. Biochem Pharmacol. 2021;187. 10.1016/j.bcp.2020.114394 33388283

[259] Pinto-Espinoza C, Guillou C, Rissiek B, Wilmes M, Javidi E, Schwarz N, et al. Effective targeting of microglial P2X7 following intracerebroventricular delivery of nanobodies and nanobody-encoding AAVs. Front Pharmacol [Internet]. 2022;13. Available from: https://pubmed.ncbi.nlm.nih.gov/36299894/10.3389/fphar.2022.1029236 PMC9589454 36299894

[260] Abad C, Demeules M, Guillou C, Gondé H, Zoubairi R, Tan YV, et al. Administration of an AAV vector coding for a P2X7-blocking nanobody-based biologic ameliorates colitis in mice. J Nanobiotechnol [Internet]. 2024;22. Available from: https://pubmed.ncbi.nlm.nih.gov/38212782/10.1186/s12951-023-02285-4 PMC10785547 38212782

[261] Demeules M, Scarpitta A, Abad C, Gondé H, Hardet R, Pinto-Espinoza C, et al. Evaluation of P2X7 receptor function in tumor contexts using rAAV vector and nanobodies (AAVnano). Front Oncol [Internet]. 2020;10. Available from: https://pubmed.ncbi.nlm.nih.gov/33042812/10.3389/fonc.2020.01699 PMC7518291 33042812

[262] Demeules M, Scarpitta A, Hardet R, Gondé H, Abad C, Blandin M, et al. Evaluation of nanobody-based biologics targeting purinergic checkpoints in tumor models in vivo. Front Immunol [Internet]. 2022;13. Available from: https://pubmed.ncbi.nlm.nih.gov/36341324/10.3389/fimmu.2022.1012534 PMC9626963 36341324

[263] Fischer T, Rotermund N, Lohr C, Hirnet D. P2Y1 receptor activation by photolysis of caged ATP enhances neuronal network activity in the developing olfactory bulb. Purinergic Signal [Internet]. 2012;8:191–8. 22187118 10.1007/s11302-011-9286-z PMC3350580

[264] Kim SS. Manipulation of P2X receptor activities by light stimulation. Mediat Inflamm [Internet]. 2016;2016. Available from: https://pubmed.ncbi.nlm.nih.gov/26884649/10.1155/2016/7852168 PMC4739260 26884649

[265] Wang T, Ulrich H, Semyanov A, Illes P, Tang Y. Optical control of purinergic signaling. Purinergic Signal [Internet]. 2021;17:385–92. 34156578 10.1007/s11302-021-09799-2 PMC8410941

[266] Zemelman BV, Nesnas N, Lee GA, Miesenböck G. Photochemical gating of heterologous ion channels: remote control over genetically designated populations of neurons. Proc Natl Acad Sci USA [Internet]. 2003;100:1352–7. 12540832 10.1073/pnas.242738899 PMC298776

[267] Grote A, Boldogkoi Z, Zimmer A, Steinhäuser C, Jabs R. Functional characterization of P2X3 receptors fused with fluorescent proteins. Mol Membr Biol [Internet]. 2005;22:497–506. 16373321 10.1080/09687860500370638

[268] Jeschik N, Schneider T, Meier C. Photocaged and mixed photocaged bioreversible-protected ATP derivatives as tools for the controlled release of ATP. Eur J Org Chem [Internet]. 2020;2020:6776–89. 10.1002/ejoc.202001229.

[269] Palinski W, Monti M, Camerlingo R, Iacobucci I, Bocella S, Pinto F, et al. Lysosome purinergic receptor P2X4 regulates neoangiogenesis induced by microvesicles from sarcoma patients. Cell Death Dis [Internet]. 2021;12:1–13. 34404763 10.1038/s41419-021-04069-w PMC8371002

[270] Gao ZG, Hechler B, Besada P, Gachet C, Jacobson KA. Caged agonist of P2Y1 and P2Y12 receptors for light-directed facilitation of platelet aggregation. Biochem Pharm [Internet]. 2007;75:1341. 18199424 10.1016/j.bcp.2007.10.037 PMC2367217

[271] Taura J, Nolen EG, Cabré G, Hernando J, Squarcialupi L, López-Cano M, et al. Remote control of movement disorders using a photoactive adenosine A2A receptor antagonist. J Control Release [Internet]. 2018;283:135–42. 29859955 10.1016/j.jconrel.2018.05.033 PMC6098950

[272] Habermacher C, Martz A, Calimet N, Lemoine D, Peverini L, Specht A, et al. Photo-switchable tweezers illuminate pore-opening motions of an ATP-gated P2X ion channel. Elife. 2016;5. 10.7554/eLife.11050 PMC4739762 26808983

[273] Peralta FA, Balcon M, Martz A, Biljali D, Cevoli F, Arnould B, et al. Optical control of PIEZO1 channels. Nat Commun [Internet]. 2023;14:1–13. 36882406 10.1038/s41467-023-36931-0 PMC9992513

[274] Lemoine D, Habermacher C, Martz A, Méry PF, Bouquier N, Diverchy F, et al. Optical control of an ion channel gate. Proc Natl Acad Sci USA [Internet]. 2013;110:20813–8. 24297890 10.1073/pnas.1318715110 PMC3870725

[275] Browne LE, Nunes JPM, Sim JA, Chudasama V, Bragg L, Caddick S, et al. Optical control of trimeric P2X receptors and acid-sensing ion channels. Proc Natl Acad Sci USA [Internet]. 2014;111:521–6. 24367083 10.1073/pnas.1318582111 PMC3890850

[276] Jabs R, Matthias K, Grote A, Grauer M, Seifert G, Steinhäuser C. Lack of P2X receptor mediated currents in astrocytes and GluR type glial cells of the hippocampal CA1 region. Glia [Internet]. 2007;55:1648–55. 17849469 10.1002/glia.20580

[277] Paoletti P, Ellis-Davies GCR, Mourot A. Optical control of neuronal ion channels and receptors. Nat Rev Neurosci [Internet]. 2019;20:514–32. 31289380 10.1038/s41583-019-0197-2 PMC6703956

[278] Li P, Rial D, Canas PM, Yoo JH, Li W, Zhou X, et al. Optogenetic activation of intracellular adenosine A2A receptor signaling in the hippocampus is sufficient to trigger CREB phosphorylation and impair memory. Mol Psychiatry [Internet]. 2015;20:1339–49. 25687775 10.1038/mp.2014.182 PMC4539301

[279] Darcey T, Taylor-Clark T, Hooper S, Kim SH. Decoding the role of genetically-distinct sensory nerves in controlling cardiopulmonary reflexes. Physiology [Internet]. 2024;39. Available from: https://journals.physiology.org. 10.1152/physiol.2024.39.S1.1867

[280] Galvan A, Caiola MJ, Albaugh DL. Advances in Optogenetic and Chemogenetic Methods to Study Brain Circuits in Non-human Primates. J Neural Transm [Internet]. 2017;125:547. 28238201 10.1007/s00702-017-1697-8 PMC5572535

[281] Kramer RH, Mourot A, Adesnik H. Optogenetic pharmacology for control of native neuronal signaling proteins. Nat Neurosci [Internet]. 2013;16:816–23. 23799474 10.1038/nn.3424 PMC4963006

[282] Emiliani V, Entcheva E, Hedrich R, Hegemann P, Konrad KR, Lüscher C, et al. Optogenetics for light control of biological systems. Nat Rev Methods Prim [Internet]. 2022;2. Available from: https://pubmed.ncbi.nlm.nih.gov/37933248/10.1038/s43586-022-00136-4 PMC10627578 37933248

