# Supplementary material for: Methodological guidelines for P2X receptor assays and data interpretation
Source: Cell Death Dis. 2026 Apr 20;17(1):521. doi: 10.1038/s41419-026-08730-0 (PMC13230957; doi:10.1038/s41419-026-08730-0)
Supplement: Supplementary file 1 — Supplementary materials [file 41419_2026_8730_MOESM1_ESM.docx]

**Gorecki et al.,**

**SUPPLEMENTARY FIGURES**


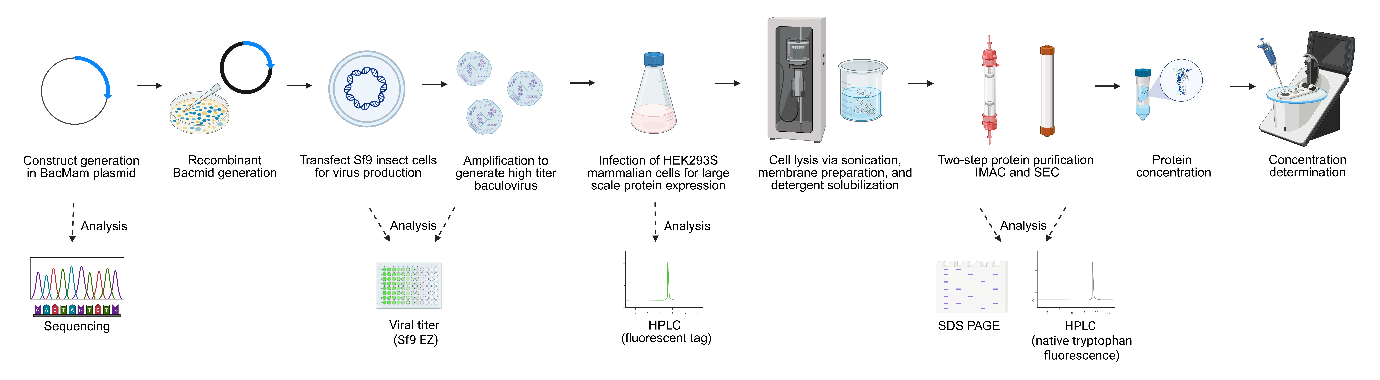


**Figure S1: A flowchart protocol for the expression, detergent solubilization/reconstitution, and purification of P2X receptors for biochemical and structural studies.**

The expression construct (a P2XR fusion protein with an EGFP and octa-histidine affinity tag incorporated at one terminus of the P2XR protomer, separated by a protease cleavage sequence) is designed in the BacMaM vector, a plasmid which is transformed into DH10Bac *Escherichia coli* to generate recombinant bacmid DNA. The bacmid DNA is then used to transfect Sf9 insect cells to produce BacMaM virus. The BacMaM virus is amplified in Sf9 insect cells to obtain a sufficient titer used to infect HEK293S mammalian cells in suspension for recombinant P2XR expression. Following cell lysis, the membranes are isolated and solubilized by the addition of detergent, and the P2XR is isolated by immobilized metal affinity chromatography (IMAC). Purity is analyzed with SDS-PAGE electrophoresis and the best fractions are pooled. Protease is added to the purified protein to remove the EGFP and affinity tag, and size exclusion chromatography (SEC) is used to separate and isolate trimeric P2XR from free EGFP and affinity tag. The highest quality SEC fractions are identified by fluorescence size exclusion chromatography (FSEC) measuring intrinsic tryptophan fluorescence, pooled together and concentrated for biochemical studies. This figure was created using [BioRender.com](https://biorender.com/).


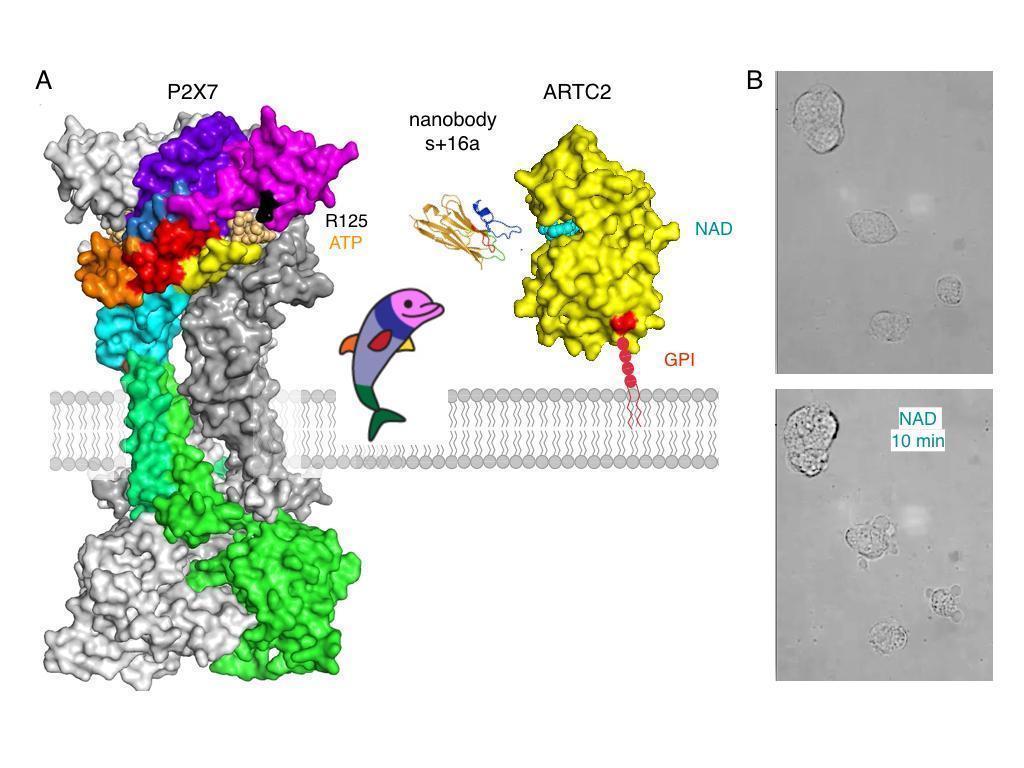


**Figure S2. ADP-ribosylation modulates P2X7 receptor function**

A) 3D models depicting the P2X7R, ARTC2 and the ARTC2-blocking nanobody s+16a. P2X7R is composed of three dolphin-shaped monomers with bound ATP and Arginine 125 highlighted in orange and black, respectively. ARTC2 is a GPI-anchored ectoenzyme that catalyses NAD-dependent ADP-ribosylation of P2X7 at R125. B) HEK cells co-transfected with mouse P2X7k and ARTC2 before (top) and 10 min after addition of NAD (bottom). Gating of P2X7R by ADP-ribosylation has resulted in extensive blebbing.


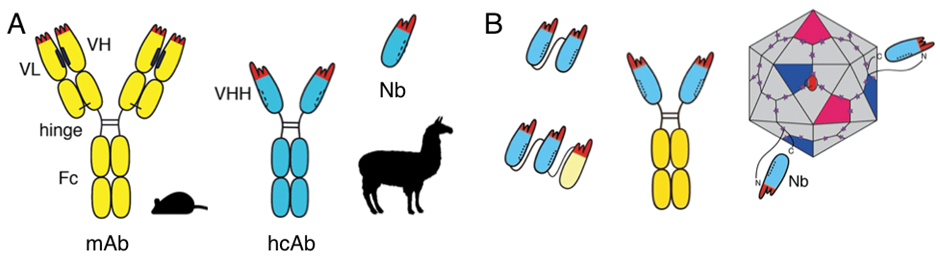


**Figure S3. Using antibodies and nanobodies to modulate P2X7 receptor functions**

A) P2X7R-specific monoclonal antibodies (mAb) are selected from immunized mice, heavy chain antibodies (hcAbs) and nanobodies (Nb) from immunized llamas. B) Stable soluble nanobodies can readily be fused to other nanobodies, the Fc region of any immunoglobulin isotype or the VP1 capsid protein of AAV gene therapy vectors.


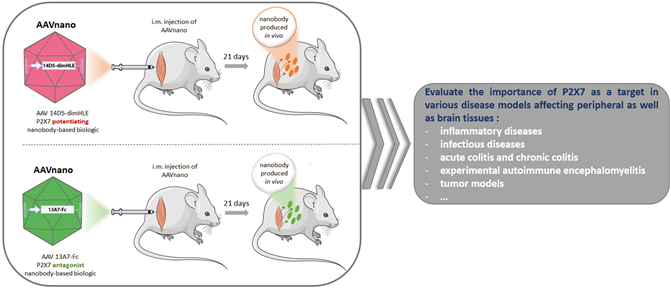


**Figure S4.: Applications of AAVnano to modulate P2X7 receptor function *in vivo* and to study its contribution in various disease models**

AAV vector coding for nanobody-based biologics (AAVnano) can be used to study the role of P2X7R *in vivo* in any animal models. For that, an AAV coding for the 13A7-Fc blocking, or the 14D5-DimHLE biologics can be injected to respectively inhibit or potentiate P2X7R function *in vivo*. Upon a single intramuscular AAV-injection, the anti-P2X7R biologics are secreted by the transduced muscle cells and reach the peripheral as well as brain tissue. Twenty-one days post AAV transduction, a time-point where functional modulation of P2X7R has reach its maximum level, disease induction/progression can be followed to determine the contribution of P2X7R *in vivo*, in acute as well as in chronic models, up to 120 days post AAV-injection.
